# C26 Cancer-Induced Muscle Wasting Is IKKβ-Dependent and NF-kappaB-Independent

**DOI:** 10.1371/journal.pone.0087776

**Published:** 2014-01-29

**Authors:** Evangeline W. Cornwell, Azadeh Mirbod, Chia-Ling Wu, Susan C. Kandarian, Robert W. Jackman

**Affiliations:** Department of Health Sciences, Boston University, Boston, Massachusetts, United States of America; McGill University, Canada

## Abstract

Existing data suggest that NF-kappaB signaling is a key regulator of cancer-induced skeletal muscle wasting. However, identification of the components of this signaling pathway and of the NF-κB transcription factors that regulate wasting is far from complete. In muscles of C26 tumor bearing mice, overexpression of dominant negative (d.n.) IKKβ blocked muscle wasting by 69% and the IκBα-super repressor blocked wasting by 41%. In contrast, overexpression of d.n. IKKα or d.n. NIK did not block C26-induced wasting. Surprisingly, overexpression of d.n. p65 or d.n. c-Rel did not significantly affect muscle wasting. Genome-wide mRNA expression arrays showed upregulation of many genes previously implicated in muscle atrophy. To test if these upregulated genes were direct targets of NF-κB transcription factors, we compared genome-wide p65 binding to DNA in control and cachectic muscle using ChIP-sequencing. Bioinformatic analysis of ChIP-sequencing data from control and C26 muscles showed very little p65 binding to genes in cachexia and little to suggest that upregulated p65 binding influences the gene expression associated with muscle based cachexia. The p65 ChIP-seq data are consistent with our finding of no significant change in protein binding to an NF-κB oligonucleotide in a gel shift assay, no activation of a NF-κB-dependent reporter, and no effect of d.n.p65 overexpression in muscles of tumor bearing mice. Taken together, these data support the idea that although inhibition of IκBα, and particularly IKKβ, blocks cancer-induced wasting, the alternative NF-κB signaling pathway is not required. In addition, the downstream NF-κB transcription factors, p65 and c-Rel do not appear to regulate the transcriptional changes induced by the C26 tumor. These data are consistent with the growing body of literature showing that there are NF-κB-independent substrates of IKKβ and IκBα that regulate physiological processes.

## Introduction

Cachexia is a metabolic condition associated with many chronic diseases and it is characterized by the severe wasting of skeletal muscle and adipose tissue which cannot be reversed by increasing caloric intake [1]. Cancer cachexia is seen in the majority of advanced cancers but it is most commonly seen in cancers of the lung and upper GI tract [1]. Cancer patients with cachexia have a lower quality of life due to compromised immune function, insulin resistance, weakness, and fatigue [2]. Patients with even mild cachexia cannot tolerate chemotherapy treatment, which further exacerbates their illness [3]. Cachexia is estimated to contribute to the deaths of approximately 30% of cancer patients [4]. For all the seriousness of this affliction, treatments remain palliative. Since the more severe consequences of cachexia seem to reside with skeletal muscle loss [1], developing a better understanding of the cellular signaling mechanisms and gene regulation in muscle could yield novel therapeutic strategies for treatment of this condition.

Research on different types of cancer has suggested a role for NF-κB signaling and NF-κB transcriptional regulation in muscle wasting [5], [6], [7]. Inhibition of the classical NF-κB pathway, by overexpression of the IkappaBalpha super repressor (IκBαSR), blocked muscle wasting in Lewis lung carcinoma (LLC) tumor-bearing mice by 50% [5]. The prototypical NF-κB transcription factor, p65 (Rel A), showed increased binding in an ELISA-based NF-κB oligonucleotide assay in muscle from mice with LLC. The increased binding was reversed when IκBαSR was overexpressed in muscle, suggesting that p65 is a NF-κB transcription factor involved in muscle wasting due to cancer, at least in mice with LLC [5]. Phosphorylation of p65 at Serine 536, thought to increase transcriptional activity, is increased in muscle from C26 tumor bearing mice [6] and in muscle from humans with pancreatic cancer [8]. On the other hand, some results on the role of NF-κB transcription factors in cachexia have been equivocal. For instance, gel shift assays showed small increases in binding of transcription factors to an NF-κB oligonucleotide in muscles from C26 tumor bearing mice [6], [9], and in another study there was no increase in protein binding to an NF-κB oligonucleotide in cachectic rats with Yoshida ascites hepatoma [10]. It is possible that different types of cancer induce muscle wasting via different cellular mechanisms. Nevertheless, only one NF-κB signaling protein (IκBα) and one NF-κB transcription factor (p65) have been studied in cancer-induced muscle wasting, and, we do not know the atrophy inducing genes that are targeted by NF-κB transcription factors.

The purpose of the present study was to identify the known mammalian NF-κB signaling proteins and NF-κB transcription factors that regulate muscle wasting due to cancer. A second purpose was to identify the NF-κB target genes on a genome-wide level in order to determine if an NF-κB transcriptional network regulates cancer-induced muscle wasting. We tested whether NF-κB signaling proteins are required for muscle wasting by overexpressing dominant negative (d.n.) forms of upstream NF-κB kinases. These are the inhibitor of kappaB kinase alpha (IKKα), IKKβ, and the NF-κB inducing kinase (NIK). The role of IκBα was tested by overexpression of a trans-dominant super repressor form of IκBα (IκBαSR). Whether the NF-κB transcription factors Rel A (p65) or c-Rel are required for muscle wasting was tested by overexpression of d.n. p65 or d.n. c-Rel, respectively. NF-κB transcription factor binding to a NF-κB oligonucleotide and NF-κB transcriptional activity of a reporter were measured in cachectic muscles. To determine if the canonical NF-κB transcription factor, p65, bound target genes that could be related to atrophy regulation, we performed ChIP-sequencing in muscle from control and tumor bearing mice in conjunction with measurement of global gene expression as a functional measure of cachexia-mediated gene expression.

This is the first in-depth study on the role of NF-κB signaling and of NF-κB transcription factors in the regulation of muscle wasting due to cancer. Although inhibition of IκBα, and particularly IKKβ activity, significantly reversed muscle wasting, there was no effect on fiber wasting due to inhibition of IKKα or NIK. Surprisingly, overexpression of dominant negative p65 or c-Rel showed little or no effect on muscle fiber wasting. Furthermore, there was no change in NF-κB transcription factor binding in a gel shift assay or in NF-κB reporter activity. Importantly, the upregulation of atrophy or cytokine genes found by gene expression microarrays was not mediated by p65-containing transcription factors as assessed by ChIP-seq. Our findings are consistent with those of Cai et al. [5] showing a role for IκBα in cancer wasting, and for the first time we show that IKKβ is required for the development of cancer cachexia, but IKKα and NIK are not. Our in-depth analysis of NF-κB signaling and transcription during cancer cachexia shows that the IKKβ is regulating gene expression by transcription factors other than NF-κB, at least in skeletal muscle during C26 cancer cachexia. These results reveal an unexpected but important facet of the regulation of cancer-induced muscle wasting.

## Methods

### Ethics Statement

This study was carried out in strict accordance with the recommendations in the Guide for the Care and Use of Laboratory Animals of the National Institutes of Health. The protocol was approved by the Charles River Campus Boston University Institutional Animal Care and Use Committee (protocol number: 12-016). All surgery was performed under ketamine/xylazine anesthesia, and all efforts were made to minimize suffering. All personnel working with animals have undergone mandatory IACUC training and yearly review: http://www.bu.edu/orctraining/animal/lacf/.

### Cells

C26 adenocarcinoma cells (National Cancer Institute, Frederick, MD) were plated and maintained at 37°C, 5% CO_2_ in Dulbecco's Modified Eagle Medium, high glucose (Invitrogen, Carlsbad, CA, USA) supplemented with 10% fetal bovine serum (Invitrogen), 1% penicillin-streptomycin (Invitrogen), and 1% L-glutamine (Invitrogen). Prior to inoculation into mice, C26 cells were trypsinized and washed twice with phosphate buffered saline and then re-suspended in a 1∶1 solution of 1X PBS and matrigel (BD Biosciences, San Jose, CA, USA).

### Animals

Eight-week-old, male CDF1 mice purchased from Charles River Laboratories (Wilmington, MA, USA) were used for all experiments. Mice were treated in strict accordance with the recommendations in the Guide for the Care and Use of Laboratory Animals of the National Institutes of Health. Animals were maintained on a 12∶12 L/D cycle, were housed individually, and were given access to food and water *ad libitum*. After three days of acclimation, animals were randomly assigned to a non-tumor control group or a C26 tumor-bearing group. Control animals received an inoculum of 150 µL 1× PBS/matrigel (1∶1) subcutaneously into the right and left flanks. Tumor-bearing animals received an inoculum of 5.0×10^5^ C26 cells suspended in 150 µL PBS/matrigel (1∶1) injected subcutaneously into the right and left flanks; this was performed on lightly anesthetized mice (40 mg/kg ketamine +5 mg/kg xylazine).

### Plasmid DNA injection in muscle

Plasmid DNA was isolated using the QIAGEN Endotoxin-Free Mega Kit as per manufacturer's instructions. Plasmid DNA was injected into muscle 10 days following the tumor inoculations, as described previously [11]. Briefly, the plasmid DNA was ethanol-precipitated and re-suspended in 0.45% sterile saline/ddH_2_O. All mice receiving surgery were given prophylactic treatments with 0.1 mg/kg Buprenorphine injection prior to surgery. Mice were anesthetized with ketamine (80 mg/kg) and xylazine (10 mg/kg). The tibialis anterior (TA) muscles from each animal were injected with 35 µg of expression plasmid in 25 µL of 0.45% sterile saline using a 29-gauge insulin syringe. Following the plasmid injection, muscles were stimulated with a low voltage electrical current using two-paddle electrodes [12] that delivered 6 pulses at 86 V/cm for 20 ms with an interpulse interval of 200 ms (Electro Square Porator ECM 830, BTX). This is a low-dose electroporation protocol that does not induce damage [13], [14], [15]. At day 25 post-inoculation, TA muscles were removed from both control and C26 tumor-bearing mice, weighed, and either snap-frozen in liquid nitrogen for biochemical analysis or mounted on tongue depressors, embedded in tissue-freezing medium, and frozen in liquid nitrogen-cooled isopentane for histochemical analysis. Injection of the NF-κB reporter plasmid was done on the same day as tumor cell inoculation. Injection of d.n.p65-EGFP was done on day 6 after tumor inoculation.

### Plasmids

The expression plasmids used were the following: d.n. IKKα-EGFP, d.n. IKKβ-EGFP, IκBαSR-EGFP, d.n. NIK-GFP, d.n. p65-EGFP, and d.n. c-Rel-EGFP. The d.n. IKKα-EGFP (K44M) and d.n. IKKβ-EGFP (K44M) expression plasmids encode EGFP fusion proteins of kinase-dead forms of IKKα and IKKβ that we have described in detail [11]. We also constructed the IκBαSR-EGFP fusion plasmid that we have described in detail [16]. The d.n. p65 (313) is a truncated form of p65 missing the C-terminal transactivation domains and was a gift from D. Guttridge [17]. We created a d.n. p65-EGFP fusion plasmid by cloning the N-terminal 313 amino acid-coding DNA sequence of p65 into the HindIII-SalI sites of pEGFP-N1 (313+238 = 551 a.a.). We verified the cloning by sequencing the plasmid and tested the dominant negative function in C2C12 myotubes, in which the d.n. p65-EGFP was able to block the TNFα activation of an NF-κB reporter by >90% (Figure S1A). For the creation of a d.n. c-Rel-EGFP plasmid, we used a mouse c-Rel template, a gift from T. Gilmore, copying the nucleic acid sequence for the Rel homology domain of the gene containing a.a. 1-288 (missing the two N-terminal transactivation domains) and adding restriction sites to clone the fragment in-frame into the EcoRI-KpnI sites of pEGFP-N1. The resulting d.n. c-Rel-EGFP insert was checked by sequencing and functioned as a dominant negative Rel because its expression reduced NF-κB reporter levels in C2C12 myotubes (Figure S1B). The d.n. NIK-GFP expression plasmid, a gift from A. Kumar, encodes a kinase dead form of NIK and EGFP from a separate cistron [18].

### Immunohistochemistry

TA muscle fiber cross-sectional areas of plasmid-transfected fibers (expressing green fluorescence fusion proteins) were compared to fiber areas in the same fields that did not take up the plasmid in both tumor-bearing and control mice. As previously described [11], frozen sections were taken from the mid-belly of the TA (7.5 µm-thick), mounted on Tissue Tack microscope slides (Polysciences, Inc, Warrington, PA, USA) and fixed in 4% paraformaldehyde. Cross sections were washed, blocked in 10% BSA and incubated with rabbit anti-laminin 1∶200 (L9393; Sigma-Aldrich) in 1% BSA followed by a Texas Red-X goat anti-rabbit IgG fluorescent dye-conjugated secondary antibody 1∶200 (T-6391; Invitrogen). Sections were mounted on a coverslip with Vectashield mounting medium (Vector Laboratories, Burlingame, CA, USA). Images were visualized at 20X magnification using an inverted light microscope (Nikon Eclipse TS 100). All images were captured with a SPOT RT camera (Diagnostic Instruments, Sterling Heights, MI, USA). Fiber cross-sectional area was calculated using the Meta-Morph Imaging System (Universal Imaging, Glendale, WI, USA). The average number of fibers measured per muscle was 200.

### Isolation of crude nuclear extracts

Crude nuclear extracts from hind limb muscles of 13 to 23 day C26-inoculated CD2F1 male mice and age matched controls were isolated according to Kumar and Boriek [19] with some modifications. All isolation steps and spins were carried out at 4°C using chilled buffers. Flash frozen muscles were suspended at 1 mg muscle weight per 18 µl of buffer (10 mM HEPES [pH 7.9], 10 mM KCl, 3 mM MgCl2, 0.1 mM EDTA [pH 8], 0.1 mM EGTA, 0.1% Triton X-100, 1 mM dithiothreitol, 0.5 mM phenylmethylsulfonyl fluoride, and protease inhibitor cocktail) and immediately homogenized on ice. Homogenized muscles were incubated on ice for 5 min followed by 20 s of vigorous vortex. Nuclei were spun at 3000× *g* for 10 min. The supernatant was removed and stored at −80°C. Nuclear pellets were washed with 1 mL of modified cytoplasmic extract buffer without Triton X-100 and centrifuged at 3000× *g* for 3 min. Pellets were resuspended in 4 µl of nuclear extract buffer (20 mM HEPES [pH 7.9], 420 mM NaCl, 1 mM EDTA, 1 mM EGTA, 25% glycerol, 1 mM dithiothreitol, 0.5 mM phenylmethylsulfonyl fluoride, and protease inhibitor cocktail) per mg of original muscle weight. Re-suspended nuclear pellets were incubated on ice for 30 min with 15 s of vigorous vortexing every 10 min. Samples were centrifuged at 16,000× *g* for 20 min and nuclear extract (supernatant) was removed and stored at −80°C.

### Electrophoretic mobility shift assay (EMSA)

Protein concentration of crude muscle nuclear extracts was determined using the Bradford assay (Bio-Rad Protein Assay Dye Reagent, Bio-Rad Laboratories, Hercules, CA, USA). Double stranded NF-κB IRDye 700 infrared dye end-labeled oligonucleotide, 5′-AGTTGAGGGGACTTTCCCAGGC-3′ (underline indicates NF-κB binding site), was purchased from LI-COR Biosciences (Lincoln, NE, USA). Consensus unlabeled competitor NF-κB oligonucleotide was purchased from Integrated DNA Technologies, Inc. (Coralville, IA, USA). EMSA binding reaction was performed using Odyssey EMSA Buffer Kit (LI-COR Biosciences) with some modifications. Total reaction volume was prepared in 20 µl. Binding reaction reagents were added to purified water in the following order: 2 µl of binding buffer (100 mM Tris, 500 mM KCl, 10 mM DTT; pH 7.5), 2 µl of 25 mM DTT/2.5% Tween-20, 1 µg/µl poly dI•dC in 10 mM Tris, 1 mM EDTA; pH 7.5, 50 fmole NF-κB IRDye 700 infrared dye end-labeled oligonucleotide, and 15–20 µg nuclear extract. In reactions with unlabeled NF-κB competitor, unlabeled and labeled oligonucleotides were mixed prior to addition to the binding reaction. Binding reactions were gently mixed and incubated for 25 min, at room temperature, in the dark. Prior to loading samples, 2 µl of Orange Loading Dye (65% w/v sucrose, 10 mM Tris-HCl pH 7.5, 10 mM EDTA, 0.3% w/v Orange G) was added to the 20 µl reaction and gently mixed.

DNA-protein complexes were separated from free oligonucleotide on non-denaturing 5% Tris-borate-EDTA, PAGE Ready Gels (Bio-Rad Laboratories, Inc.). Samples were electrophoresed at 76 V for 2 hours at room temperature in the dark. Protein bound to labeled oligonucleotide complexes were visualized using an Odyssey Infrared Imager (LI-COR Biosciences).

### NF-κB-dependent reporter activity

At the indicated time points, TA muscles transfected with an NF-κB-dependent luciferase reporter were harvested and then homogenized in 1 mL Passive Lysis Buffer (Promega, Madison, WI) with protease and phosphatase inhibitors (Roche Applied Science, Indianapolis, IN). Homogenates were centrifuged at 5,000× *g* at 4°C for 20 minutes. Supernatant was collected and mixed with Luciferase Assay Reagent (Promega) according to manufacturer's instructions. Luciferase activity was measured by a TD-20/20 luminometer (Turner Designs, Sunnyvale, CA, USA).

### Western blot analysis

Protein concentration of muscle lysates in passive lysis buffer was determined using the Bradford assay (Bio-Rad Protein Assay Dye Reagent, Bio-Rad Laboratories, Hercules, CA). Fifty to 75 micrograms of protein was denatured and size fractionated on 4–15% SDS-polyacrylamide gels. Proteins were transferred to an Immobilon-FL polyvinylidene fluoride membrane (Millipore, Bedford, PA, USA), blocked in Odyssey blocking buffer (LiCor Biotechnology, Lincoln, NE, USA) and incubated with the appropriate primary antibody according to the manufacturer's instructions. Antibodies included: anti-IKKα/IKK1 (IMG-5477, Imgenex, San Diego, CA, USA), IKKβ/IKK2 (#2684), NF-κB2 (p100/p52; #4882), and p-IκBα (#9246) (Cell Signaling Technology, Danvers, MA, USA), IκBα (sc-371), NF-κB p65 (sc-7151), c-Rel (sc-6363), and NIK (sc-7211) (Santa Cruz Biotechnology, Santa Cruz, CA, USA). Anti-GAPDH (G-9545) was used as a loading control (Sigma-Aldrich, St. Louis, MO, USA). Alexa Fluor 680 fluorescent dye-conjugated secondary antibody (Invitrogen) was used for visualization with the Li-Cor Odyssey infrared imaging system described previously [20].

### RNA isolation

Gastrocnemius and plantaris muscles were harvested from anesthetized control and tumor-bearing mice (25 days post-inoculation), snap-frozen in liquid nitrogen, and stored at -80°C until further processing. Total RNA was extracted using Trizol reagent (Invitrogen, Carlsbad, CA, USA) according to the manufacturer's instructions. The extracted total RNA was further purified using an RNase-Free DNase I kit (Qiagen, Valencia, CA, USA) and an RNeasy Mini kit (Qiagen) as previously described [21]. Extracted RNA was quantitated by UV spectrophotometry and quality checked by a 1% denaturing agarose gel. Samples were also verified by Bioanalyzer 2100 analysis and had a minimal RIN number of 8.0 (Agilent, Palo Alto, CA, USA).

### Microarray analysis

For microarray analysis, the RNA samples described above were sent to the Boston University Medical Center Microarray Core Facility for amplification, labeling, and hybridization on a mouse Affymetrix Gene 1.0 ST array (Santa Clara, CA, USA) per the manufacturer's instructions to measure the expression of 28,132 well-annotated genes. A total of 6 array images (3 control muscle samples and 3 C26 muscle samples) were acquired by GeneChip Scanner 3000 TG and quality assessed by Affymetrix Expression Console (Santa Clara, CA, USA). Gene expression signal values were generated by the Expression File Creator module of the GenePattern platform (Broad Institute, Cambridge, MA) and robust multi-array analysis algorithm (RMA) was used for data preprocessing [22]. Brainarray MoGene 1.0 ST custom CDF file v.13 was used for probe annotation [23]. Both .*cel* files and expression values were deposited into MIAME compliant NCBI Gene Expression Omnibus with accession number: GSE48363 (link: http://www.ncbi.nlm.nih.gov/geo/query/acc.cgi?token=rnupvmqqyocggpk&acc=GSE48363). Differential gene expression was computed using the Comparative Marker Selection module in GenePattern which compares mean differences between control and C26 groups by two-way parametric t-tests. Parameters used to identify genes that were differentially expressed in muscle from tumor-bearing mice were: *P*-value≤0.05, *q*-value≤0.05, and fold change greater than 1.5-fold. The up- or down-regulated gene lists were uploaded to the DAVID Bioinformatics database for functional annotation and for gene ontology assignment based on the biological process of the genes compared to that of *mus musculus* genome where the threshold of EASE score (a modified Fisher Exact P-value) was set at 0.01 and a minimum gene count of 2.

### ChIP-sequencing

Gastrocnemius and plantaris muscles were isolated from control (i.e. non-tumor-bearing) and C26 tumor-bearing mice at day 25 post tumor inoculation. Freshly dissected muscle was minced and cross-linked in 1% formaldehyde for 15 minutes, quenched with glycine and then frozen in liquid nitrogen. For each condition (i.e., control or C26) plantarflexor muscle from four legs were pooled, homogenized, and chromatin isolated as we detailed previously [21]. This material was sonicated to yield chromatin fragments that were on average 250 bp. An aliquot of sonicated chromatin was put aside to be used as the input fraction for ChIP-PCR. The rest of the chromatin was diluted in IP buffer and split into a group for incubation with the p65 antibody (Santa Cruz SC-372X) and a group without any primary antibody; this was done for chromatin from both control and C26 groups. Samples with and without primary antibody were incubated for 16 hrs at 4°C with constant low speed mixing and then the antibody-chromatin complexes were captured with Protein G magnetic beads. The chromatin was eluted from the beads and crosslinks reversed, followed by pronase/RNase treatment and precipitation of the DNA. One tenth of the material was used for PCR for a gene already shown to have robust p65 binding in order to test the p65 ChIP. Three different DNA libraries were made: control p65 ChIP, C26 p65 ChIP, and ChIP with no primary antibody. The libraries were prepared for high throughput sequencing using the Illumina ChIP-seq Library kit. An aliquot of each library was examined by acrylamide electrophoresis and Sybr-gold staining to estimate the quality by size and intensity of the product which appears as a smear with an average size of 350 bp. The entire procedure for making the three libraries was repeated a second time with new muscle samples in order to have two ChIP-seq libraries for each of the three groups. The libraries were sent to The Whitehead Institute (Cambridge, MA) where they were cleaned of adapter dimers using Ampure XL beads. The cleaned libraries were tested by Bioanalyzer and qPCR quality control was performed in order to determine how much of each library to use. The libraries were sequenced using Illumina Solexa sequencing on a GA II sequencer. The resulting sequences from control and C26 samples were aligned to the mouse genome (mm9 version) using ELAND. The sequences were sent to our lab in the ELAND format.

The alignments for the two independent control samples were pooled and the same was done for the two C26 samples. In each case this was done by combining the .bam forms of the alignments on the Galaxy website. The sequences were then evaluated with the FastQC package of quality control tests, which was obtained as a standalone program from the Brabaham Institute (http://www.bioinformatics.babraham.ac.uk/projects/fastqc/). After the initial FastQ analysis two operations were performed on the ChIP-seq data: 1. we removed pcr duplicates using samtools rmdup via Galaxy, and 2. again using samtools, we took advantage of the mapping labels in the ELAND data to isolate only reads mapped at unique sites in the mouse genome. Sequence reads were 35 or 36 base pairs. The three files of ChIP-seq data were: control p65 ChIP (10.7 million reads), C26 p65 ChIP (11.1 million reads), and a file representing background sequence reads (8.5 million) obtained by combining control and C26 sonicated chromatin which was run through the ChIP without a primary antibody (no antibody group). These three post-QC sequence .bam files are available online at: (https://drive.google.com/folderview?id=0B0Ph0YwXvamQektEaTFkcElSLTQ&usp=sharing) The aligned sequences were converted to .sam format and then uploaded to the peak finder program in ChIPseeqer [24] with the following parameters: 1. threshold  = 5, 2. fold change  = 2 or greater (compared to background), 3. fragment length  = 250 bp, 4. peak length at least 100 bp and 5. peak separation at least 100 base pairs. In addition to identification of control and C26 p65 peaks relative to the background (no antibody), ChIPseeqer identified the gene region of the p65 binding peaks (i.e., genomic location of the peaks).

The sets of control and C26 p65 peaks were run on PeakAnalyzer [25] (http://www.ebi.ac.uk/research/bertone/software#peakanalyzer) in order to find the gene with the nearest TSS for each peak. This generates a list of genes with peaks less than 100 kb from the TSS. The lists were then compared to the lists of increased and decreased mRNA expression from the control vs. C26 microarray data by using a perl algorithm located on a web page developed by Jim Lund at the University of Kentucky (http://nemates.org/MA/progs/Compare.html).

### Positive Control for ChIP

To confirm the correct binding of the p65 antibody we studied ChIP with PCR for two consecutive highly studied and verified NF-κB promoter binding sites in the mouse IP-10 (Cxcl10) gene [26]. This ChIP experiment also used an antibody for p50 (Santa Cruz SC-1190X). A positive control for ChIP was made with chromatin from C2C12 myotubes that had been treated for 4 hours with 10 ng/ml of mouse TNFα. We previously showed that this treatment strongly induced a NF-κB luciferase reporter and increased IP-10 mRNA levels, both of which are dependent on p65 [27]. For the test experiment we used methods described in our previous work [21]. Briefly, formaldehyde fixed chromatin from untreated and TNFα-treated C2C12 myotubes was immunoprecipitated with p65 antibody, p50 antibody, or no antibody and the Protein G-captured precipitates were processed to produce DNA which was tested with PCR using primers that flank the two consecutive NF-κB sites in the IP-10 gene. In addition, we also evaluated p65 and p50 binding to the IP-10 promoter using the chromatin from control and C26 muscles.

### ChIP-PCR for Fbxo6

ChIP-PCR was performed from muscle tissue fixed, homogenized and sonicated as for ChIP-seq, with the exception that p65 antibody was attached to the Protein G magnetic beads by pre-incubation(overnight at 4C) before incubation with the chromatin, and the DNA products were used as template for PCR with primers flanking target binding site of the Fbxo6 gene, as determined from ChIP-seq. The target site from the ChIP-seq was in the first intron of the Fbxo6 gene, 2 kb downstream of the transcription start site. The sequences of the primers used for the PCR were agctcgttgatgtggaccat (left primer) and cctcctgctttaggctcctt (right primer) and produced a PCR product of 302 bp.

### Statistics

An ANOVA or a *t*-test was used for analysis, depending on the number of groups being compared. Data are expressed as means ± SE, and significance was established at *P*<0.05. Statistics for microarray data are described in the microarray section.

## Results

### Characterization of C26 tumor-bearing mice

Characterization of this tumor model in our lab involved the inoculation of CDF1 mice with C26 tumor cells in PBS/matrigel (n = 164) or with PBS/matrigel only (n = 142). Mice were sacrificed at 12, 13, 14, 17, 18, 19, 21, 22, 23, 24, 25, and 26 days post tumor cell inoculation. In severe cachexia (days 23–26 post-inoculation) tumor-bearing mice lost 22.1%±0.8% of initial body mass while the non-tumor controls gained 12.3%±0.7% of initial body mass (Figure S2A). As tumor mass increased tibialis anterior (TA) muscle mass decreased (FigureS S2B). The TA muscle mass of severely cachectic mice was 40.4±0.3 mg compared to 51.1±0.3 mg for age-matched control mice. Tumor-bearing mice in severe cachexia had 214±8.7 mg of epididymal fat pad mass while control mice had 465±10 mg (Figure S2C). The spleen mass of tumor-bearing mice was 205±4.6 mg compared to 75.5±0.7 mg in non-tumor-bearing mice (Figure S2D). Tumor mass increased as a function of time post-inoculation (Figure S2E).

### Effect of dominant negative NF-κB signaling proteins on muscle fiber atrophy in C26 tumor-bearing mice

**Figure 1:**
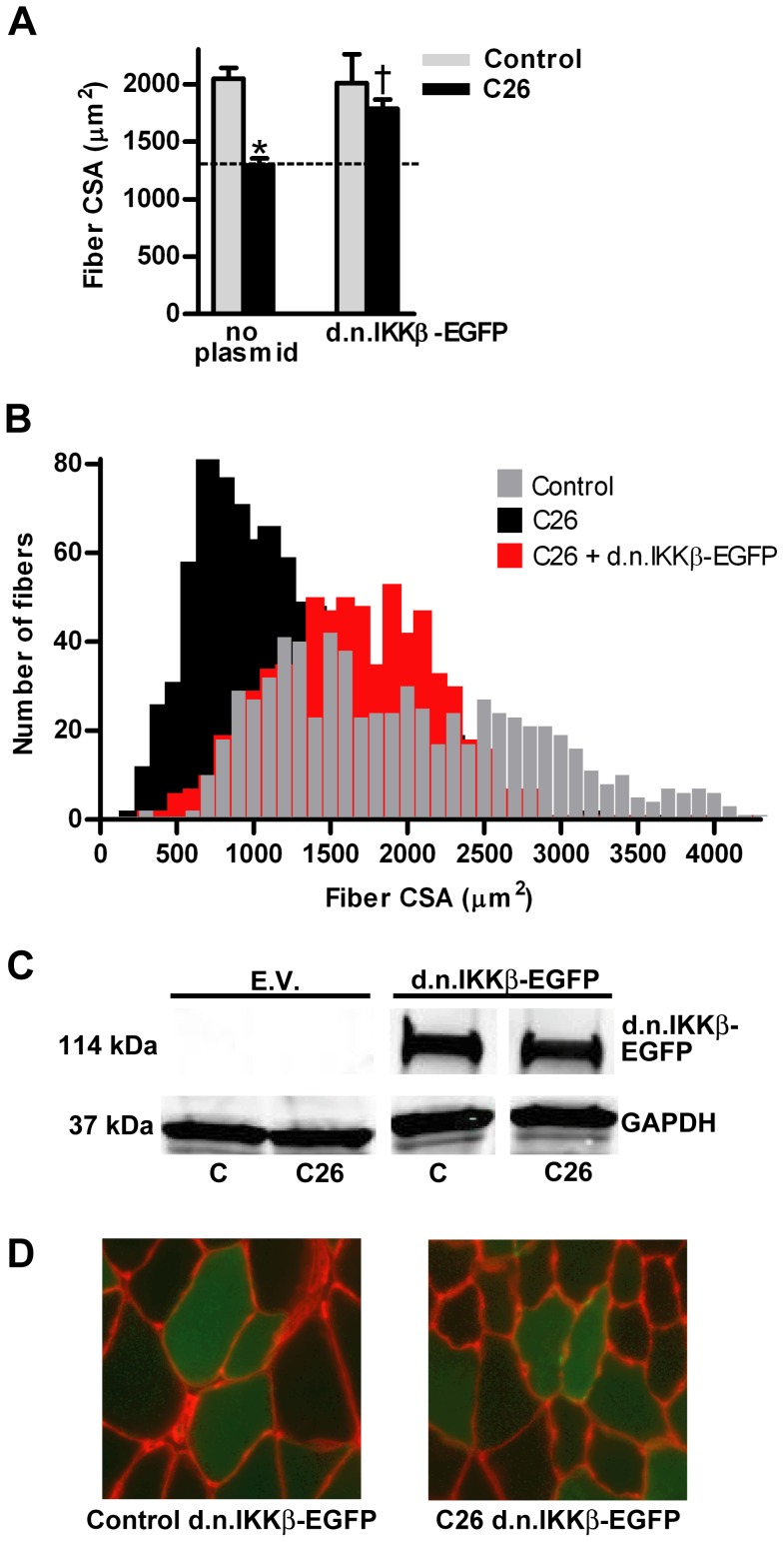
Effects of d.n. IKKβ-EGFP on muscle fiber cross sectional area (CSA). (A) Mean tibialis anterior (TA) fiber cross-sectional area in control and C26 tumor-bearing mice in the presence or absence of d.n. IKKβ-EGFP. Each bar represents mean fiber area from 8 muscles (± SE). The average number of fibers measured per muscle was 200. (B) Fiber area frequency distribution of all fibers from muscles of control, C26, and C26 + d.n. IKKβ-EGFP groups. (C) Western blots of lysates from TA muscles injected with the empty vector (EV) or d.n. IKKβ-EGFP plasmid confirms overexpression of the fusion protein. d.n. IKKβ-EGFP is 114 kDa. All samples are from the same immunoblot. (D) Representative cross sections of TA muscles from control and C26 tumor-bearing mice injected with the d.n. IKKβ-EGFP plasmid. Fluorescent red represents laminin staining (anti-laminin incubation followed by Texas Red-X fluorescent dye-conjugated secondary antibody). Fluorescent green cytoplasm represents expression of transfected d.n. IKKβ-EGFP. C  =  control; * significantly different from control (*P*<0.05). † significantly different from C26 fibers not transfected with d.n. IKKβ-EGFP (*P*<0.05).

**Figure 2:**
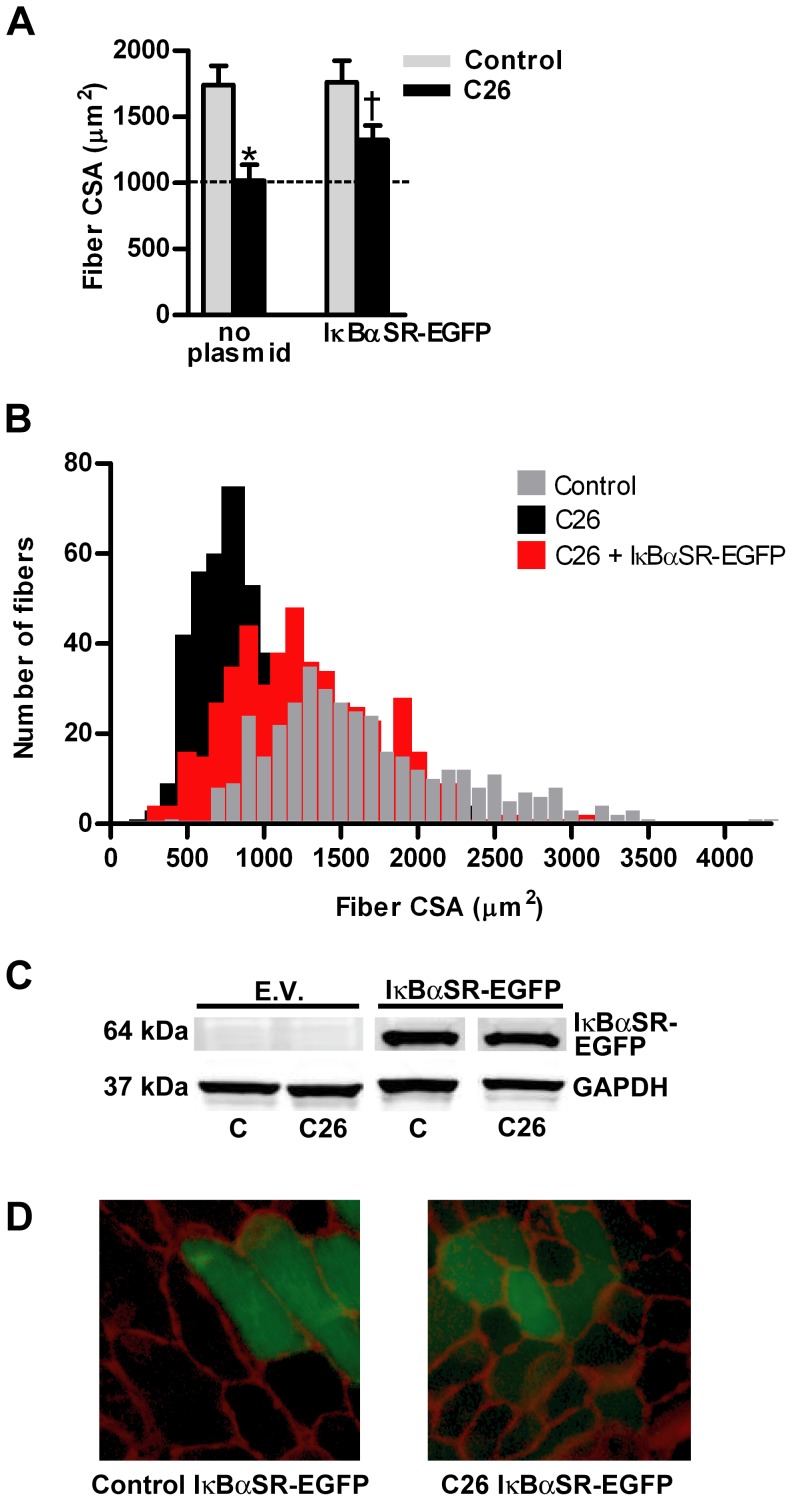
Effects of IκBαSR-EGFP on muscle fiber cross sectional area (CSA). (A) Mean tibialis anterior (TA) fiber cross-sectional area in control and C26 tumor-bearing mice in the presence or absence of IκBαSR-EGFP. Each bar represents mean fiber area from 8 muscles (± SE). (B) Fiber area frequency distribution of all fibers from muscles of control, C26, and C26 + IκBαSR-EGFP groups. (C) Western blots of lysates from TA muscles injected with the empty vector (EV) or IκBαSR-EGFP plasmid confirms overexpression of the fusion protein. IκBαSR-EGFP is 64 kDa. All samples are from the same immunoblot. (D) Representative cross sections of TA muscles from control and C26 tumor-bearing mice injected with the IκBαSR-EGFP plasmid. C  =  control. * significantly different from control (*P*<0.05). † significantly different from C26 fibers not transfected with IκBαSR-EGFP (*P*<0.05).

**Figure 3:**
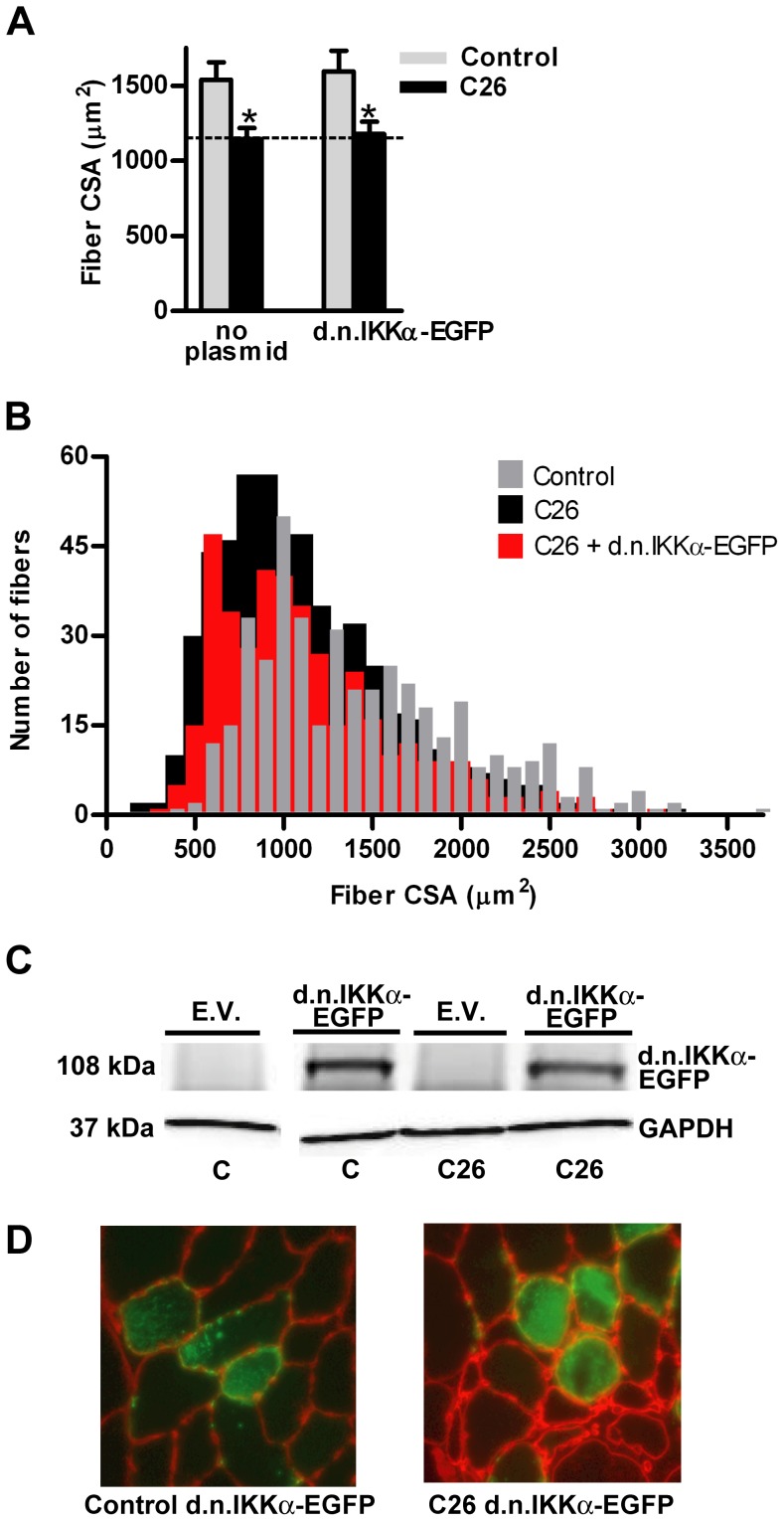
Effects of d.n.IKKα-EGFP on muscle fiber cross sectional area (CSA). (A) Mean tibialis anterior (TA) fiber cross-sectional area in control and C26 tumor-bearing mice in the presence or absence of d.n. IKKα-EGFP. Each bar represents mean fiber area from 8 muscles (± SE). (B) Fiber area frequency distribution of all fibers from muscles of control, C26, and C26 + d.n. IKKα-EGFP groups. (C) Western blots of lysates from TA muscles injected with the empty vector (EV) or d.n. IKKα-EGFP plasmid confirms overexpression of the fusion protein. d.n. IKKα-EGFP is 108 kDa. All samples are from the same immunoblot. (D) Representative cross sections of TA muscles from control and C26 tumor-bearing mice injected with the d.n. IKKα-EGFP plasmid. C  =  control. * significantly different from control (*P*<0.05).

**Figure 4:**
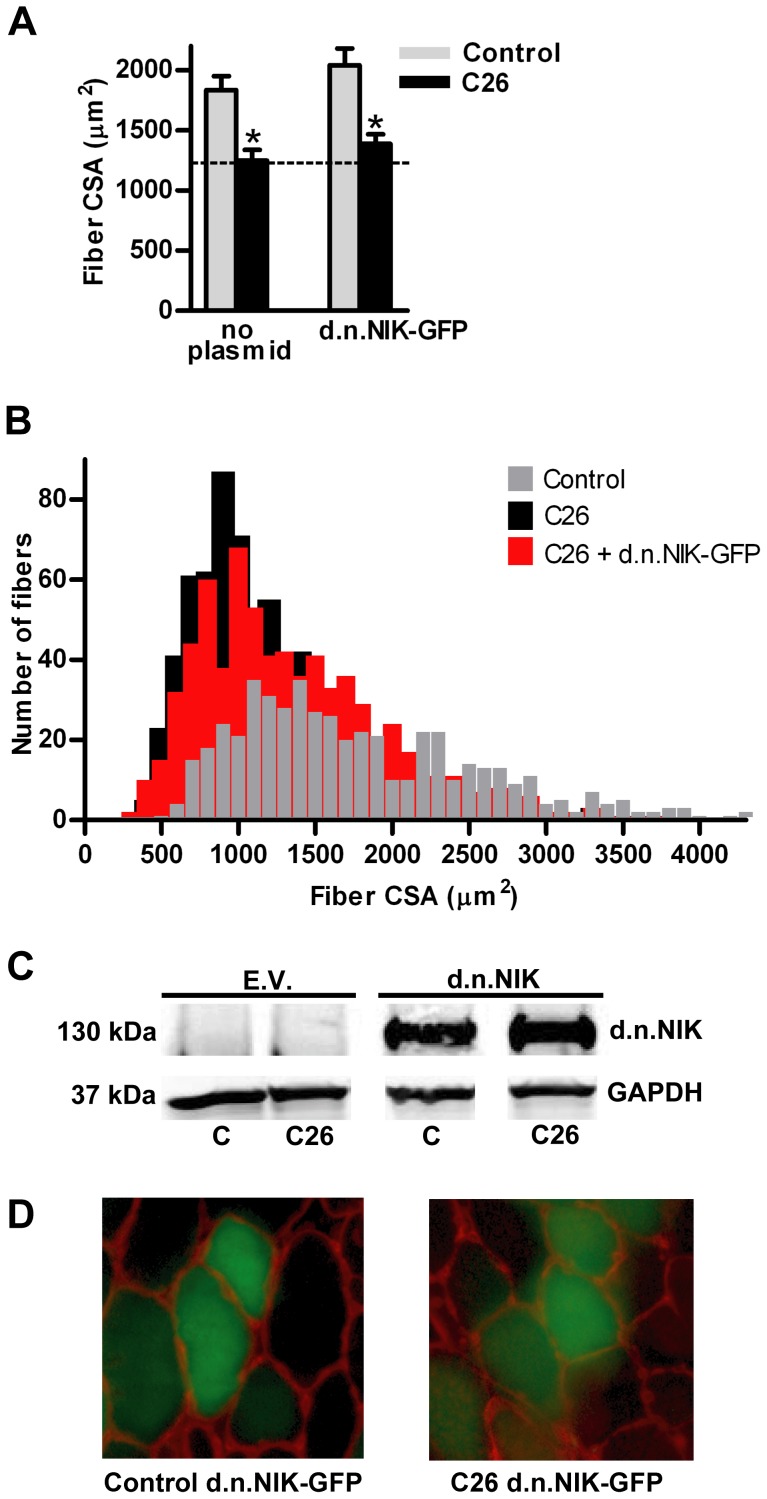
Effects of d.n. NIK on muscle fiber cross sectional area (CSA). (A) Mean tibialis anterior (TA) fiber cross-sectional area in control and C26 tumor-bearing mice in the presence or absence of d.n. NIK. Each bar represents mean fiber area from 8 muscles (± SE). (B) Fiber area frequency distribution of all fibers from muscles of control, C26, and C26 + d.n. NIK groups. (C) Western blots of lysates from TA muscles injected with the empty vector (EV) or d.n. NIK plasmid confirms overexpression of the protein. d.n. NIK = 130 kDa. All samples are from the same immunoblot. (D) Representative cross sections of TA muscles from control and C26 tumor-bearing mice injected with the d.n. NIK plasmid (encodes GFP on separate cistron). C  =  control. * significantly different from control (*P*<0.05).

The mean fiber cross sectional area from TA muscles of C26 tumor-bearing mice was 35% less than fibers from non-tumor-bearing control mice (Figure 1A). However, overexpression of d.n.IKKβ-EGFP blocked fiber atrophy by 69%. Plotting the individual fiber areas from muscles in each group by size vs. frequency reveals the heterogeneous distribution of muscle fiber area and shows the leftward shift to smaller fiber size due to cancer and the blocking of that shift in fiber size in the same muscles overexpressing d.n.IKKβ-EGFP (Figure 1B). Confirmation of expression of full-length d.n.IKKβ-EGFP is illustrated by western blot (Figure 1C), and examples of muscle sections used for measurement of fiber area are shown (Figure 1D). Overexpression of IκBαSR-EGFP blocked C26 tumor-induced fiber atrophy by 41% (Figure 2A, B). Confirmation of the overexpression of full length IκBαSR-EGFP is shown by western blot (Figure 2C) and representative muscle cross-sections from which fiber area was measured are shown (Figure 2D). Overexpression of d.n.IKKα-EGFP (Figure 3A–D) and d.n.NIK-GFP (Figure 4A–D) did not block C26 tumor-induced fiber cross sectional area atrophy. We have previously shown that overexpression of EGFP alone does not affect fiber area [16].

### Effect of dominant negative Rel A or c-Rel on muscle fiber atrophy in C26 tumor-bearing mice

**Figure 5:**
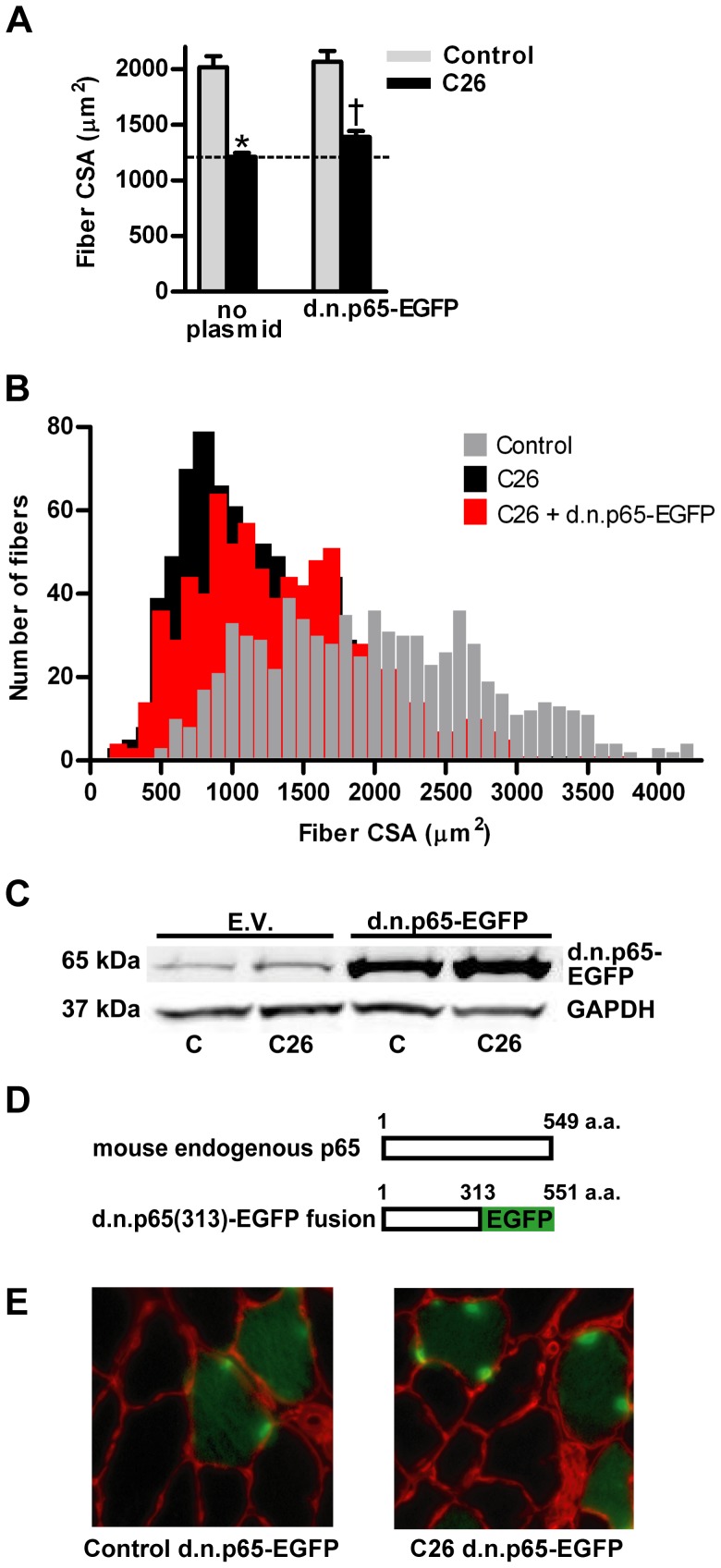
Effects of d.n.p65-EGFP on muscle fiber cross sectional area (CSA). (A) Mean tibialis anterior (TA) fiber cross-sectional area in control and C26 tumor-bearing mice in the presence or absence of d.n. p65-EGFP. Each bar represents mean fiber area from 8 muscles (± SE). (B) Fiber area frequency distribution of all fibers from muscles of control, C26, and C26 + d.n. p65-EGFP groups. (C) Western blots of lysates from TA muscles injected with the empty vector (EV) or d.n. p65-EGFP plasmid confirms overexpression of the fusion protein. (D) The d.n. p65(313)-EGFP fusion protein (313 a.a.+238 a.a. = 551 a.a. = 63 kDa) is of similar molecular weight as endogenous mouse whole p65 protein (549 a.a. = 60.2 kDa) and they are not separable on a 4-15% gradient polyacrylamide gel. All samples are from the same immunoblot. (E) Representative cross sections of TA muscles from control and C26 tumor-bearing mice injected with the d.n. p65-EGFP plasmid. C  =  control. * significantly different from control (*P*<0.05). † significantly different from C26 fibers not transfected with d.n.p65-EGFP (*P*<0.05).

**Figure 6:**
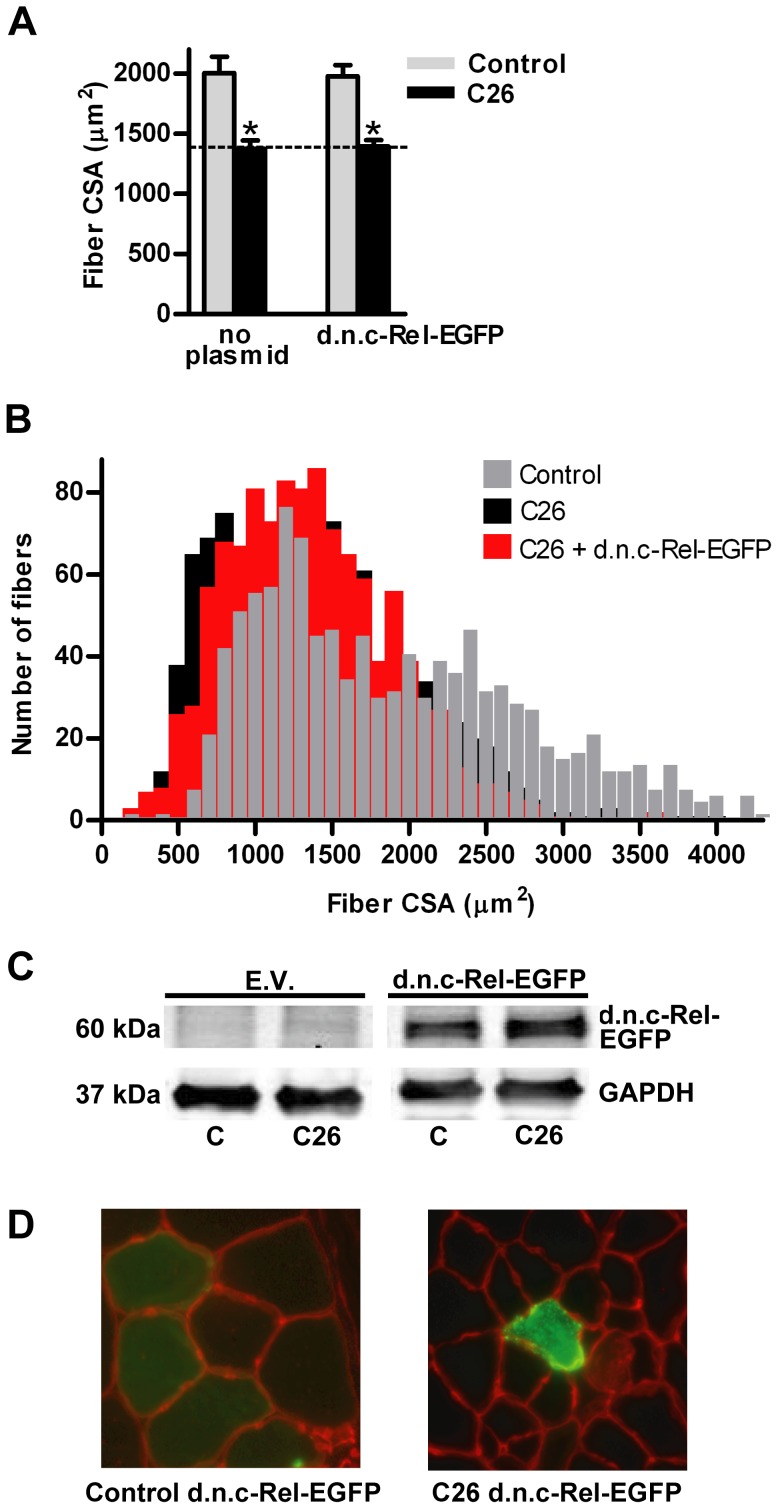
Effects of d.n. c-Rel-EGFP on muscle fiber cross sectional area (CSA). (A) Mean tibialis anterior (TA) fiber cross-sectional area in control and C26 tumor-bearing mice in the presence or absence of d.n. c-Rel-EGFP. Each bar represents mean fiber area from 8 muscles (± SE). (B) Fiber area frequency distribution of all fibers from muscles of control, C26, and C26 + d.n. c-Rel-EGFP groups. (C) Western blots of lysates from TA muscles injected with the empty vector (EV) or d.n. c-Rel-EGFP plasmid confirms overexpression of the fusion protein. d.n.c-Rel-EGFP is 60 kDa. All samples are from the same immunoblot. (D) Representative cross sections of TA muscles from control and C26 tumor-bearing mice injected with the d.n.p65-EGFP plasmid. C  =  control. * significantly different from control (*P*<0.05).

Overexpression of d.n. p65-EGFP reversed C26 fiber atrophy by only 20% (Figure 5A–D) and overexpression of d.n. c-Rel-EGFP did not affect fiber atrophy (Figure 6A–D).

### NF-κB transcription factor binding to a NF-κB oligonucleotide (gel shift assay)

**Figure 7:**
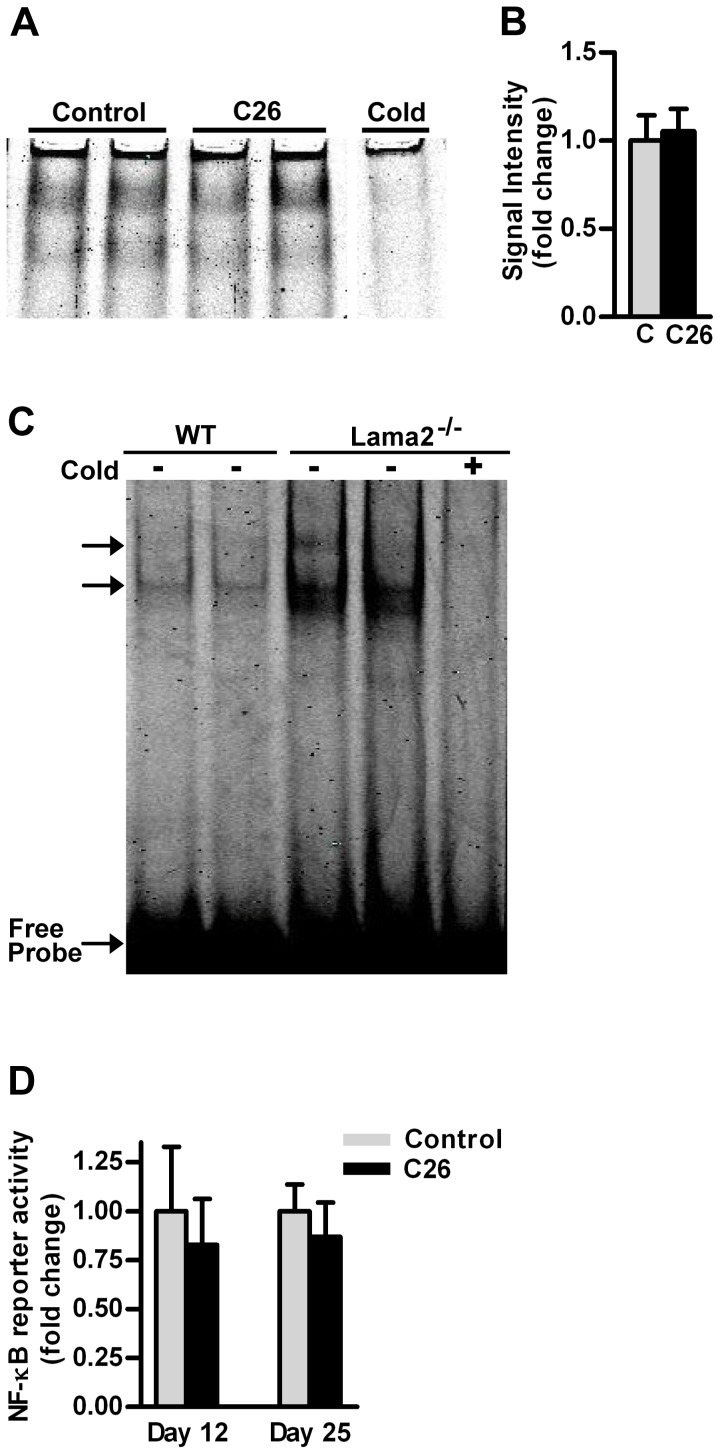
Gel shift assay of NF-κB binding and NF-κB reporter activity. (A) Bands represent protein-oligonucleotide binding of gastrocnemius nuclear extracts from control and C26 mice 23 days post-inoculation. 20 µg of protein incubated with infrared dye labeled NF-κB oligonucleotide. A lane labeled “Cold” indicates incubation with 200X unlabeled consensus oligonucleotide competitor. Competitor lane was on same gel as lanes 1 through 4. (B) Signal intensity of bands representing protein binding to NF-κB oligonucleotide; control mean value represents 12 independent samples, C26 mean value represents 14 independent samples. Values were combined from 13, 17 and 23 days post-tumor cell inoculation since there was no difference in binding compared to controls at any time point. (C) As a positive control for the NF-κB gel shift assay, nuclear extracts isolated from hind limb muscles of 42 day old *Lama2^−/−^* mice compared to aged matched C57BL/6 wild type (WT) controls are shown. 15 µg of protein incubated with infrared dye labeled NF-κB oligonucleotide. + indicates incubation with 150X unlabeled consensus oligonucleotide competitor. Each lane represents an independent muscle sample. (D) NF-κB-dependent reporter activity in TA muscles from control and C26 tumor-bearing mice. NF-κB-dependent luciferase activity in TA muscles at 12 and 25 days after tumor cell inoculation representing mild and severe cachexia. The number of muscles per group was 6 for the 12-day time point and 8 for the 25-day time point.

Gel shift assays were performed using gastrocnemius muscle nuclear extracts isolated from tumor-bearing mice (13–23-day post-inoculation) and from age matched controls (12 control muscles and 14 C26 muscles). Figure 7A shows representative examples of NF-κB oligonucleotide binding from control and C26 extracts isolated at 23 days post-tumor cell inoculation. The specificity of protein-DNA binding was determined by addition of excess unlabeled consensus NF-κB oligonucleotide competitor to binding reaction. There was no significant difference in the amount of protein binding due to the tumor regardless of the number of days after tumor cell inoculation. Measurement of band intensity was combined for each time point and mean values were plotted for control vs. C26 mice (Figure 7B).

As a positive control for our method of nuclear extract isolation and NF-κB gel shift procedures, we performed a gel shift assay in muscles from control and laminin-alpha2 deficient mice (muscles from M. Girgenrath). These muscles are characterized by inflammatory markers including significant mononuclear infiltration [28] and accordingly we found significant increases in protein binding (Figure 7C) to the same NF-κB oligonucleotide used for control vs. cancer mice.

### NF-κB-dependent reporter activity in muscles from control vs. C26 mice

Another assay to determine if NF-κB transcriptional activity was elevated in muscles from tumor bearing mice was measurement of the activity of a NF-κB dependent luciferase reporter plasmid injected into the TA muscle of control and C26 mice. Two time points post-inoculation were assessed which represented mild and severe cachexia (12 and 25 days). NF-κB reporter activity in the TA muscle was not different at either 12 or 25 days indicating that protein binding and/or post-translational changes to protein binding of the reporter gene did not increase NF-κB transcriptional activity in cachexia (Figure 7D). We have shown that this same NF-κB reporter plasmid is strongly activated in mouse hind limb muscle due to unloading [16], [29] and in cultured mouse myotubes due to treatment with TNFα, IL-1β, IL-1α, or TWEAK [27], [30]. These results showing no activation of the NF-κB reporter in muscles of mice with C26 tumors are consistent with the gel shift data.

### Expression of p100/p52 in muscles from control vs. C26 mice

When the alternative NF-κB signaling pathway is activated, p100 is phosphorylated and post-translationally processed to the p52 NF-κB transcription factor. Western blot showed no change in the amount of p100 or p52 protein in muscles from control vs. C26 mice 21 days after tumor cell inoculation (Figure S3). This is consistent with the lack of effect on cachexia with overexpression of d.n. NIK and d.n. IKKα which are upstream kinases of the alternative NF-κB pathway.

### Genome-wide mRNA expression microarray

**Figure 8:**
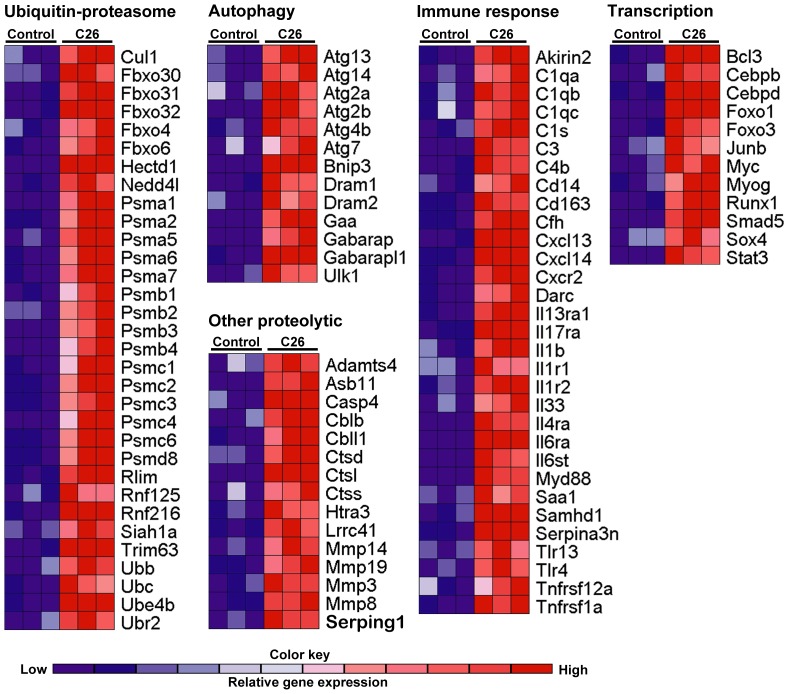
Heatmaps of selected upregulated genes in plantar flexor muscles from several functional categories. Data are from muscles at 25 days post-tumor inoculation (severe cachexia). Red color in the heatmap represents gene upregulation in muscles from C26 mice compared to the lower control values represented by blue. All genes represented are increased greater than 1.5-fold. Genes involved in: ubiquitin-proteasomal protein degradation, autophagy, other proteolytic processes (e.g. caspases, cathepsins, and metalloproteases), immune responses (innate and adaptive), and transcription factor genes are shown. The heatmaps capture most of the upregulated genes in these functional categories but do not represent a complete list (see Table S2). Each color block represents one pooled sample (control n = 3; C26 n = 3).

Whole-transcript gene expression arrays were used for mRNA expression profiling of muscles to compare with ChIP-seq data and to verify gene expression changes in our hands. Among 28,000 transcripts investigated at day 25 post tumor inoculation, 1,907 genes were differentially expressed in muscles from tumor bearing mice using q≤0.05 and fold change ≥1.5; 1034 genes were upregulated and 873 genes were down-regulated (Table S1). Differentially expressed genes in muscle from tumor bearing mice were similar to those previously published in severe C26 cancer cachexia [31] such as upregulation of acute phase response genes, ubiquitin-proteasomal degradation genes, autophagy genes, key atrophy genes including MuRF1, atrogin/MAFbx (Fbxo32), Fbxo30, Fbxo31, cathepsin L, caspase4, Foxo1, Foxo3, Stat3, Socs3, C/EBPδ, Bcl-3, myogenin, Jun B, etc.). Selected transcripts that were upregulated by 1.5-fold or greater are listed for several relevant functional categories; this includes genes involved in ubiquitin-proteasomal protein degradation, autophagy, other proteolytic processes, and immune function (Figure 8). To identify the computationally derived functional processes from the 1,907 differentially expressed genes, we used DAVID's Functional Annotation analysis (Table 1). For upregulated genes, 22 GO terms (biological process) were significantly enriched; 9 were associated with proteolysis or catabolic processes, 4 were related to inflammatory processes, and 3 were associated with translation. The genes found for these GO categories are contained in the selected upregulated genes shown in the heatmap (Figure 8). For downregulated genes, 8 GO terms were enriched; these processes were oxidation reduction, generation of precursor metabolites and energy, extracellular matrix organization and muscle development. The list of up- and down-regulated genes belonging to the DAVID-identified GO categories are listed in Table S2.

**Table 1:** Enriched GO terms in muscle of C26 mice using DAVID functional annotation.

Enriched GO terms in up-regulated genes	Count
GO:0006508: proteolysis	87
GO:0009057: macromolecule catabolic process	75
GO:0030163: protein catabolic process	64
GO:0044265: cellular macromolecule catabolic process	63
GO:0051603: proteolysis involved in cellular protein catabolic process	57
GO:0044257: cellular protein catabolic process	57
GO:0010033: response to organic substance	53
GO:0006952: defense response	52
GO:0043632: modification-dependent macromolecule catabolic process	52
GO:0019941: modification-dependent protein catabolic process	52
GO:0006396: RNA processing	48
GO:0009611: response to wounding	39
GO:0006954: inflammatory response	32
GO:0022613: ribonucleoprotein complex biogenesis	28
GO:0042254: ribosome biogenesis	26
GO:0006511: ubiquitin-dependent protein catabolic process	26
GO:0009725: response to hormone stimulus	24
GO:0045087: innate immune response	19
GO:0043434: response to peptide hormone stimulus	18
GO:0002526: acute inflammatory response	17
GO:0009894: regulation of catabolic process	12
GO:0008286: insulin receptor signaling pathway	10

Enriched GO terms in down-regulated genes	Count
GO:0055114: oxidation reduction	61
GO:0006091: generation of precursor metabolites and energy	36
GO:0043062: extracellular structure organization	24
GO:0030198: extracellular matrix organization	19
GO:0014706: striated muscle tissue development	19
GO:0060537: muscle tissue development	19
GO:0022900: electron transport chain	18
GO:0030199: collagen fibril organization	8

Count denotes the number of up/down-regulated genes associated with each GO term.

### ChIP-Seq of p65 in muscle from control vs. C26 tumor mice

**Figure 9:**
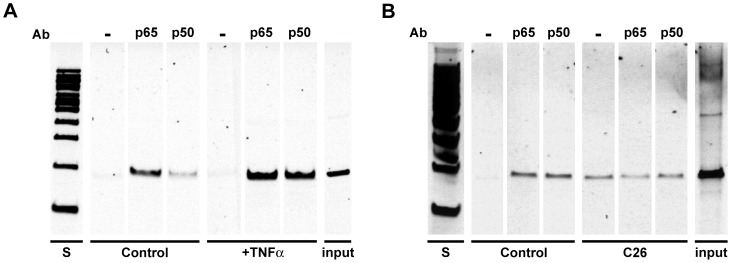
ChIP PCR to validate the p65 antibody and comparison of p65 binding in cancer cachexia vs. a classic inflammatory system. (A) Chromatin was prepared from C2C12 myotubes that had been untreated or treated with 10 ng/ml mouse TNF for 4 hours and from (B) gastrocnemius/plantaris muscles of mice with and without 25 days of C26 tumor-induced cachexia. Chromatin was sonicated and immunoprecipitated without antibody or with the same antibody to p65 used for ChIP-seq or with a p50 antibody. Captured precipitates were used to prepare DNA template evaluated by PCR with primers that flank two closely spaced validated NF-κB sites in the IP-10 promoter. PCR products are shown on acrylamide gels, stained with Sybr gold. Standards are 100 bp ladder. Input lanes are PCR products using the same primers on chromatin taken before ChIP.

p65 carries the transactivation domain for itself and for p50 in the classical heterodimer of NF-κB and so its binding in ChIP is referred to “as the hallmark of the active NF-κB complex” [32]. We tested our p65 antibody, already validated in the literature [21], [33], for use in our p65 ChIP sequencing libraries by testing it on a bona fide κB site cluster (two sites separated by 46 nucleotides) in the IP-10 gene. We prepared untreated or TNFα-treated C2C12 myotube formaldehyde-fixed chromatin and performed ChIP using no antibody, p65 antibody, or p50 antibody. Binding was evaluated by PCR using primers flanking the highly conserved verified NF-κB sites in the IP-10 gene promoter. The results showed marked increases in the PCR product representing p65 and p50 binding due to TNFα treatment (Figure 9A).

We then performed ChIP from control and C26 muscles, using our tested p65 antibody and sequenced the resulting DNA libraries. We used the quality control analyses of FastQC (Figures S4-S6) to confirm that our libraries consisted of high quality uniquely mapped sequences on the mouse genome without redundancies due to the PCR used in amplification. This generated 11.1×10^6^ and 10.8×10^6^ unique alignments for cancer and control sequences, respectively, on the mouse genome. ChIPseeqer was used set to the same threshold that we had employed for Bcl-3 binding to unloaded muscle chromatin [34] and peaks were identified from the p65 antibody-captured alignments in control and C26 muscles. Allowing each peak to be a maximum of 100 kb from its nearest gene TSS, there were only 100 peaks in the controls and 57 peaks in the C26 samples. The genes associated with these peaks are presented in Table S3. For the C26 peaks, 25 were also present in the controls, an indication of no change in binding for almost 50% of the genes. Table S3 also shows p65 peak-containing genes that were the same as genes found to be upregulated or downregulated by at least 1.5 fold in our gene expression arrays. One gene is upregulated in the C26 group and has a p65 peak in the C26 ChIP-seq but not in the control, Fbxo6, while 5 genes are downregulated in C26 and correspond to genes with control p65 peaks that were lost in C26 p65 ChIP-seq, Hdh3, Ibtk, Itgb6, Jph2 and Nav2. The Fbxo6 peak occurred in the first intron of its gene and was verified with ChIP-PCR as shown in figure S7.

## Discussion

The intracellular signaling pathways and transcriptional regulation of muscle wasting due to cancer are just beginning to be understood. The purpose of this study was to identify NF-κB signaling proteins, and NF-κB transcription factors functioning in cancer-induced muscle wasting. The existing literature indicates the involvement of the NF-κB signaling protein, IκBα, because LLC tumor-bearing mice overexpressing a super repressor form of IκBα in skeletal muscle showed a 50% inhibition of muscle fiber atrophy [5]. In that study, increased binding of the p65 NF-κB transcription factor was found in muscle from mice with tumors and this was reversed in the presence of overexpressed IκBαSR. There is some evidence that NF-κB transcription may be activated in the type of cancer used in the present study, mouse colon adenocarcinoma (C26), although NF-κB reporter activity was not measured [6]. Therefore, we performed in-depth studies to determine the requirement of the major known NF-κB signaling proteins and the NF-κB transcription factors in cancer-induced muscle wasting.

We have now shown that there is a robust, 69% inhibition of C26-induced muscle wasting when d.n. IKKβ is overexpressed, demonstrating a significant requirement of IKKβ. We also confirmed that an IκBα super repressor can lessen C26-induced wasting as previously described for LLC-induced wasting [5]. However, IKKα and NIK were not necessary for muscle wasting indicating that the alternative NF-κB signaling pathway is not involved in the atrophy process. This was further confirmed by western blots showing no change in the expression of the p52 NF-κB transcription factor or its precursor protein, p100, in C26 cancer (Figure S3). Classical and alternative NF-κB signaling pathways have been shown to have divergent roles in muscle in response to cytokine mediated atrophy and during muscle development [18], [35], [36], [37], however since we found evidence for IKKβ, and not alternative NF-κB signaling in C26 cachexia, those divergent actions do not appear to function here.

Surprisingly, neither p65 nor c-Rel was required for C26 atrophy since overexpression of dominant negative forms of these proteins did not affect fiber size significantly. Consistent with these findings, gel shift assays showed no change in nuclear protein binding to NF-κB oligonucleotides and there was no change in NF-κB-dependent reporter activity in muscles of control vs. C26 tumor bearing mice. Importantly, using genome-wide ChIP-sequencing, we found almost no NF-κB p65 binding peaks in genes shown to be upregulated in cancer cachexia as assessed by our gene expression microarrays. We recently showed that when a NF-κB transcription factor is unequivocally required for muscle atrophy, ChIP-seq is a powerful tool that further validates the requirement of the factor by means of the functional categories of genes showing transcription factor binding peaks [34]; this was not the case in C26 cancer for p65 ChIP-seq. An explanation for this appears in several papers that do find p65 binding sites by ChIP-seq in which the induction of p65 binding requires strong inflammatory mediators such as LPS [32] or TNFα [33], [38]. We show in Figure 9A that incubation with TNFα produces IP-10 p65 binding in treated C2C12 cells, however the IP-10 gene does not show increased p65 binding due to C26-mediated cachexia (Figure 9B). Corroborating this, our gene expression array for control vs. C26 muscle showed that IP-10 mRNA levels were unchanged (not in Table S1 since there was no change). C26-induced cachexia does not appear to be mediated by the kind of inflammatory cytokines that would produce p65 activation.

The lack of binding peaks on genes involved in atrophy processes could be due to our measurement of binding at only one time point, severe cachexia. However, at the time of severe cachexia our microarray data show that the expression of genes that characterize muscle wasting is ongoing, so at least p65 shows little direct involvement in activation of genes at this time. In addition, the overexpression of d.n. p65 or d.n. c-Rel would have shown a significant blocking of muscle atrophy if these NF-κB factors were required at an earlier time point in cachexia. Moreover, NF-κB reporter assays and gel shift assays were done at several time points, from 12 to 25 days post-tumor cell inoculation, and at no time was there an activation of the reporter nor a significant increase in binding to an NF-κB oligonucleotide, unlike the robust increase we showed in a primary muscle inflammatory disease (Figure 7C) or in disuse atrophy [39].

Taken together, our data show that although IKKβ is necessary for muscle wasting during cancer cachexia, the changes in gene expression shown by microarray are not regulated by NF-κB transcription factors. Another paper that found some evidence of NF-κB in C26 cachexia also reported that this signaling was not responsible for the upregulated atrophy genes [6]. The requirement for IKKβ but not NF-κB transcription factors suggests that inhibition of IKKβ is blocking other cellular processes requiring this kinase that in turn regulate muscle wasting via transcription factors other than the NF-κB family members. The possibility that the IKKs may be activated while not activating NF-κB-regulated transcription has been demonstrated multiple times over the past decade [40], [41], [42], [43], [44]. This has been referred to as IKK-dependent, NF-κB-independent signaling [41]. A number of IKK substrates besides IκBα have been identified, and, other substrates also exist for IκBα besides the Rel transcription factors [40]. We have recently reviewed this literature in detail [45]. The process of muscle wasting due to C26 cancer appears to be another example of IKKβ signaling that is independent of NF-κB transcription. As further evidence that IKKβ is not phosphorylating IκBα, levels of endogenous IκBα and IκBα phosphorylation are unchanged at either 10 or 24 days after tumor inoculation (Figure S8).

Recently, other transcription factors have been shown to play a role in cancer cachexia. C/EBPβ is required for LLC-induced muscle wasting as demonstrated using C/EBPβ knockout mice [46]. Foxo transcription factors have been shown to block LLC-induced muscle wasting [47] and C26 wasting (A. Judge, manuscript in review). Inhibition of Stat3 blocked C26-induced atrophy using several experimental approaches [48] but it is not known if Stat3 is required for LLC-induced wasting. The observation that cancer-induced muscle wasting is associated with increased p65 binding to an oligonucleotide in an ELISA format may be specific to LLC cancer [5] since none of our assays show p65 to be required for C26 cancer wasting.

In conclusion, our data show for the first time that IKKβ is required for C26 cancer-induced muscle wasting because inhibition of IKKβ blocks fiber atrophy by 69%. Also we show that the alternative (non-canonical) NF-κB signaling pathway involving IKKα and NIK is not required for muscle wasting due to C26 tumors. Interestingly, we find that NF-κB transcription is not driving the muscle wasting process as evidenced by a lack of an effect on atrophy due to genetic inhibition of the Rel proteins (p65 and c-Rel) containing transactivation domains, the lack of increased NF-κB protein binding in gel shift and reporter assays, and the lack of atrophy target gene binding of the prototypical Rel transcription factor p65 in genome-wide ChIP-seq experiments. Further studies on the role of Stat3 and FoxO in C26-induced wasting are promising areas of investigation, while the identification of their target genes using ChIP-seq is a logical next step in understanding the transcriptional control of muscle wasting. The downstream target(s) of IKKβ is also a prime avenue of research in order to further understand the cellular signaling underlying muscle wasting due to colon adenocarcinoma.

## Supporting Information

Figure S1 **Verification of the inhibiting effect of the dominant negative forms of p65 and c-Rel.** (A) A test of d.n. p65-EGFP by treatment of myotubes for 4 hours with 10 ng/ml mouse TNFα. c-Rel-mediated NF-κB activity does not respond to TNFα in C2C12 cells, but the resting level of NF-κB activity is determined in part by c-Rel as shown in B. (B) C2C12 cells transfected with a NF-κB reporter plus control (pEGFP), d. n. p65-EGFP fusion plasmid, or d.n. c-Rel-EGFP fusion plasmid.
(TIF)

Figure S2 **Characterization of cachexia in C26 tumor bearing mice**. (A) Body mass change as a function of tumor size. Each value for tumor mice has a corresponding value for age-matched control mice. (B) TA muscle mass as a function of tumor size. Values for age-matched control mice are plotted for comparison. (C) Epididymal fat mass as a function of tumor size. Values for age-matched control mice are plotted for comparison. (D) The correlation between spleen size and tumor mass. (E) Relationship between tumor size and time of inoculation.
(TIF)

Figure S3 **Western blot of p100 and p52 from TA muscle of control and C26 mice**. Muscles were from mice 21 days post C26 inoculation. 70 µg of lysate loaded per lane and blot incubated with p100/p52 (NF-κB2) antibody. Each lane represents an independent muscle sample. Extract from Hela cells used as positive control.
(TIF)

Figure S4 **FastQC printout for bam format of C26 p65 ChIP-seq**. FastQC evaluates the ChIP-seq alignment data on 11 criteria. The output in this case passed all tests and was then used for peak analysis.
(PDF)

Figure S5 **FastQC printout for bam format of Control p65 ChIP-seq**. The sequences passed all tests and were taken for peak analysis.
(PDF)

Figure S6 **FastQC printout for bam format of no antibody ChIP-seq (background)**. The background alignments passed all tests and were used as the input reference for the ChIPseeqer program.
(PDF)

Figure S7 **ChIP-PCR of the p65 binding site in the Fbxo6 gene.** Chromatin prepared exactly as that for ChIP-seq was incubated with no antibody on Protein G beads or the p65 antibody (SC-372X) which had been attached to Protein G magnetic beads and then the material was washed and prepared as for the ChIP-seq except that instead of making a sequencing library with the resulting DNAs, PCR was run on the DNA as described in the Methods section. The results are presented from a 10% acrylamide TBE gel, stained with Sybr Gold. The expected band is 302 bp and the lanes are from left to right: S the 100 bp standards; the no antibody lanes, Control and C26; the p65 lanes Control and C26 and the input lanes, Control and C26. By simple densitometry, the intensity of the C26 p65 band is 1.8 fold greater than that for the Control p65 band.
(TIF)

Figure S8 **Western blots of phospho-IκBα and IκBα in control and C26 muscle.** (A) Gastrocnemius muscle extracts from mice 10 days post inoculation. (B) Gastrocnemius muscle extracts from mice 24 days post inoculation. 60 µg protein loaded per lane. Each lane represents an independent muscle sample. There were no changes in p-IκBα or IκBα expression due to either time point of cachexia.
(TIF)

Table S1 **Differentially expressed genes due to cancer (q<0.05, p<0.05, ≥1.5-fold change).**
(XLSX)

Table S2 **Cachexia-induced up- and down-regulated genes (≥1.5-fold) belonging to DAVID-identified GO categories shown in** **Table 1** **.**
(XLSX)

Table S3 **The genes with p65 ChIP-seq peaks from control (no tumor) and C26 muscle.** From left to right the columns are: control p65, with reference to no antibody background alignments; C26 p65, with reference to no antibody background alignments; Intersect, the genes in common between control and C26; Control/Array+, the genes from Control ChIP-seq in common with >1.5 fold upregulated genes from the gene expression microarray; Control/Array-, the genes from the Control ChIP-seq in common with >1.5 fold downregulated genes from the gene expression microarray; C26/Array+, the genes from C26 ChIP-seq in common with >1.5 fold upregulated genes from the gene expression microarray; C26/Array-, the genes from the C26 ChIP-seq in common with >1.5 fold downregulated genes from the gene expression microarray.
(DOCX)
