# Supplementary figures and images for: C26 Cancer-Induced Muscle Wasting Is IKKβ-Dependent and NF-kappaB-Independent
Source: PLoS One. 2014 Jan 29;9(1):e87776. doi: 10.1371/journal.pone.0087776 (PMC3906224; doi:10.1371/journal.pone.0087776)

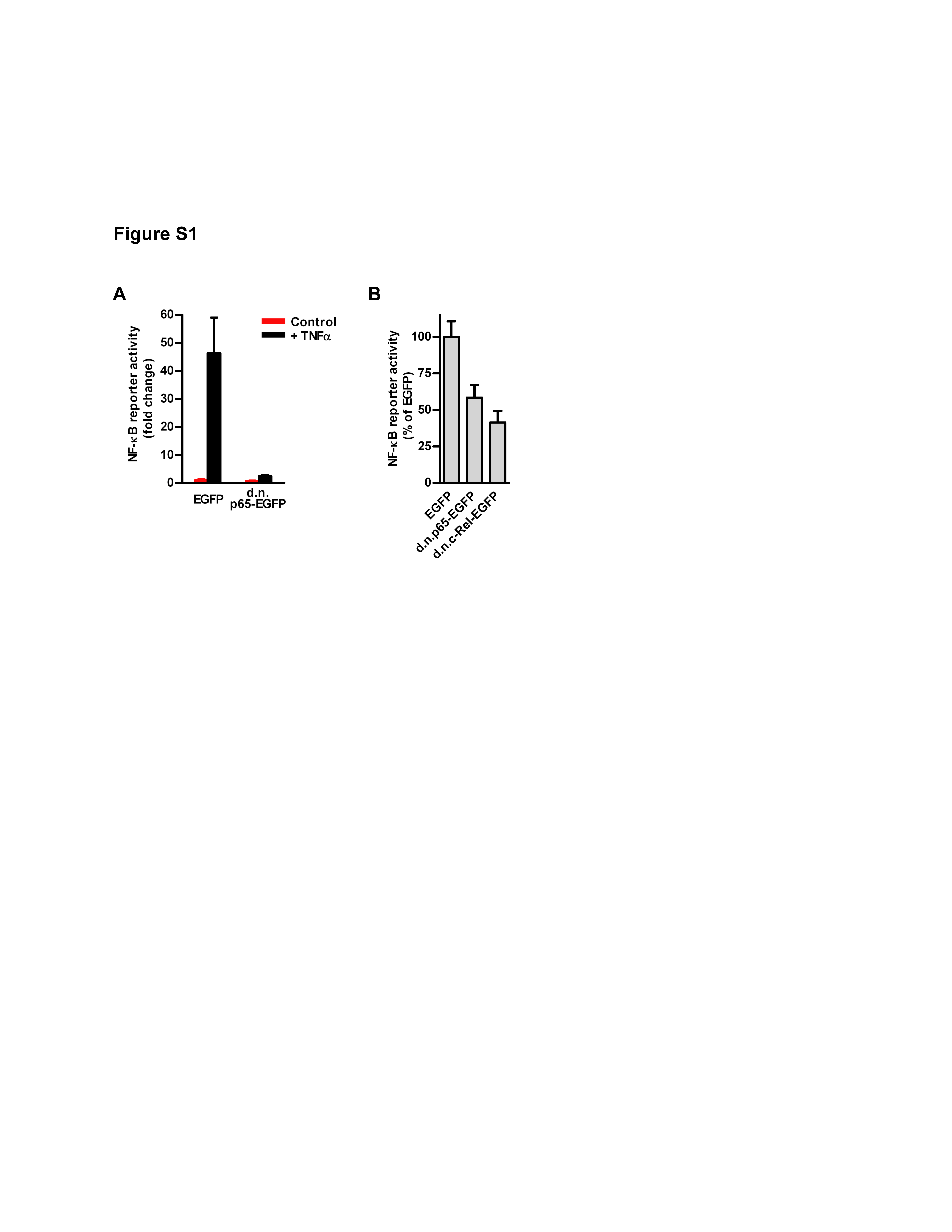

Supplement: Figure S1 — Verification of the inhibiting effect of the dominant negative forms of p65 and c-Rel. (A) A test of d.n. p65-EGFP by treatment of myotubes for 4 hours with 10 ng/ml mouse TNFα. c-Rel-mediated NF-κB activity does not respond to TNFα in C2C12 cells, but the resting level of NF-κB activity is determined in part by c-Rel as shown in B. (B) C2C12 cells transfected with a NF-κB reporter plus control (pEGFP), d. n. p65-EGFP fusion plasmid, or d.n. c-Rel-EGFP fusion plasmid. (TIF) [file pone.0087776.s001.tif]

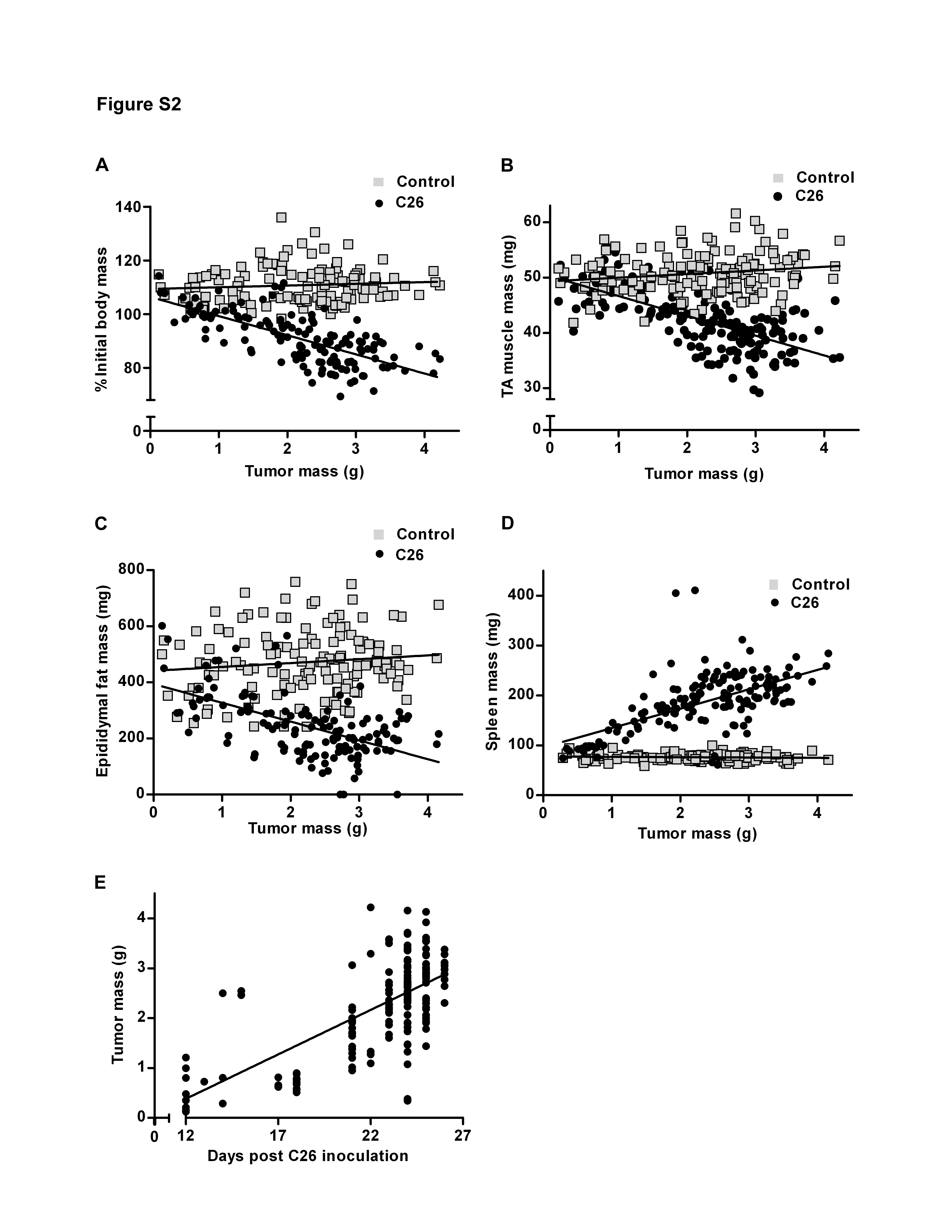

Supplement: Figure S2 — Characterization of cachexia in C26 tumor bearing mice. (A) Body mass change as a function of tumor size. Each value for tumor mice has a corresponding value for age-matched control mice. (B) TA muscle mass as a function of tumor size. Values for age-matched control mice are plotted for comparison. (C) Epididymal fat mass as a function of tumor size. Values for age-matched control mice are plotted for comparison. (D) The correlation between spleen size and tumor mass. (E) Relationship between tumor size and time of inoculation. (TIF) [file pone.0087776.s002.tif]

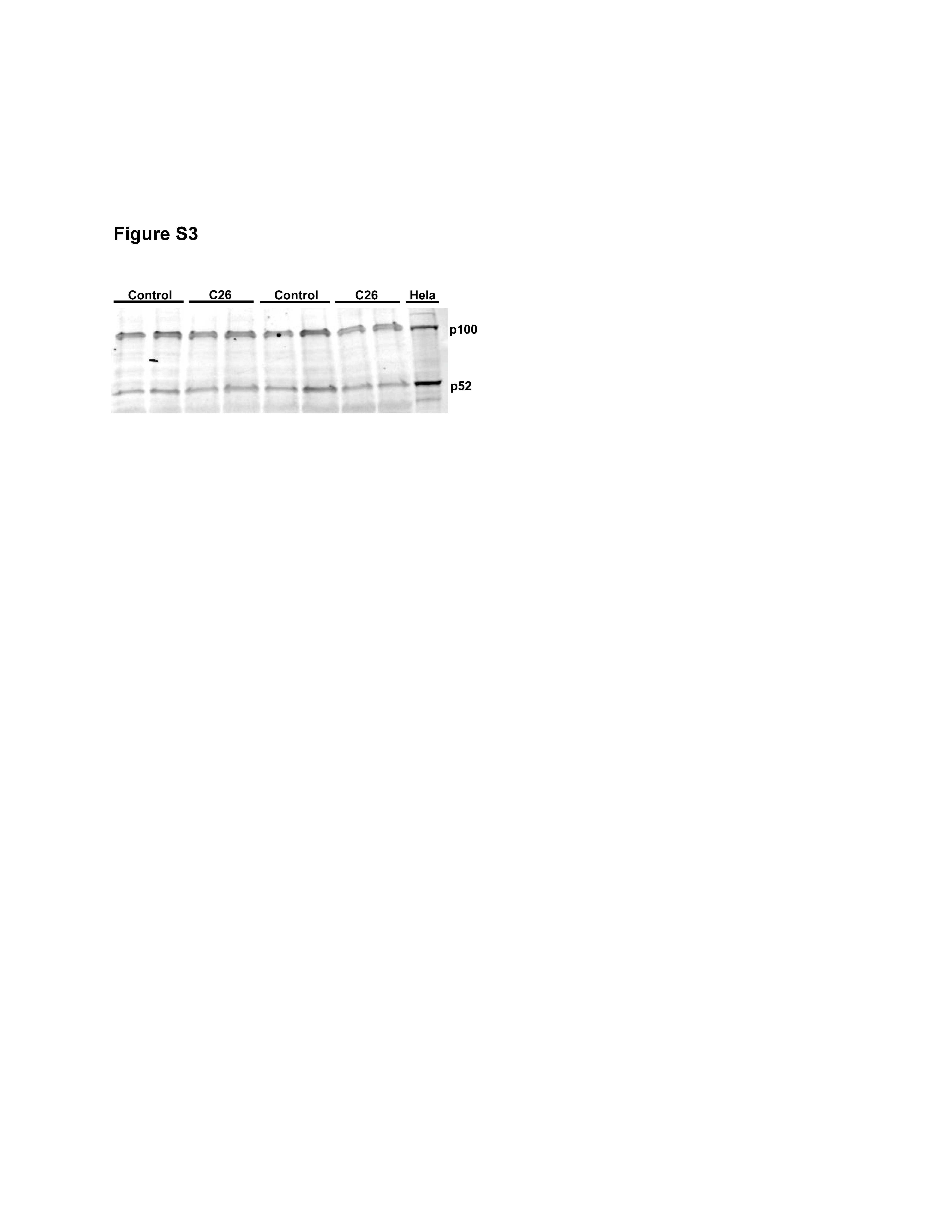

Supplement: Figure S3 — Western blot of p100 and p52 from TA muscle of control and C26 mice. Muscles were from mice 21 days post C26 inoculation. 70 µg of lysate loaded per lane and blot incubated with p100/p52 (NF-κB2) antibody. Each lane represents an independent muscle sample. Extract from Hela cells used as positive control. (TIF) [file pone.0087776.s003.tif]

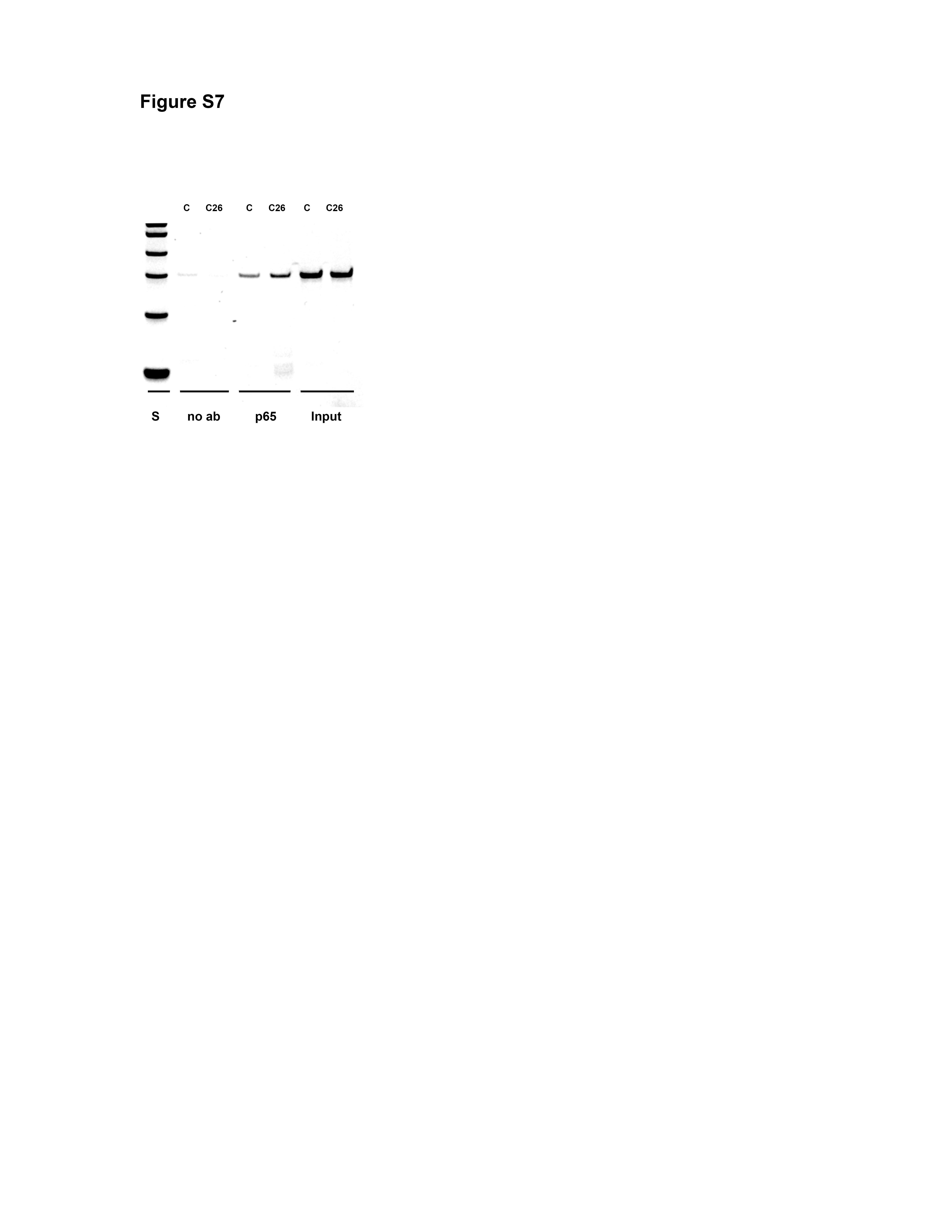

Supplement: Figure S7 — ChIP-PCR of the p65 binding site in the Fbxo6 gene. Chromatin prepared exactly as that for ChIP-seq was incubated with no antibody on Protein G beads or the p65 antibody (SC-372X) which had been attached to Protein G magnetic beads and then the material was washed and prepared as for the ChIP-seq except that instead of making a sequencing library with the resulting DNAs, PCR was run on the DNA as described in the Methods section. The results are presented from a 10% acrylamide TBE gel, stained with Sybr Gold. The expected band is 302 bp and the lanes are from left to right: S the 100 bp standards; the no antibody lanes, Control and C26; the p65 lanes Control and C26 and the input lanes, Control and C26. By simple densitometry, the intensity of the C26 p65 band is 1.8 fold greater than that for the Control p65 band. (TIF) [file pone.0087776.s007.tif]

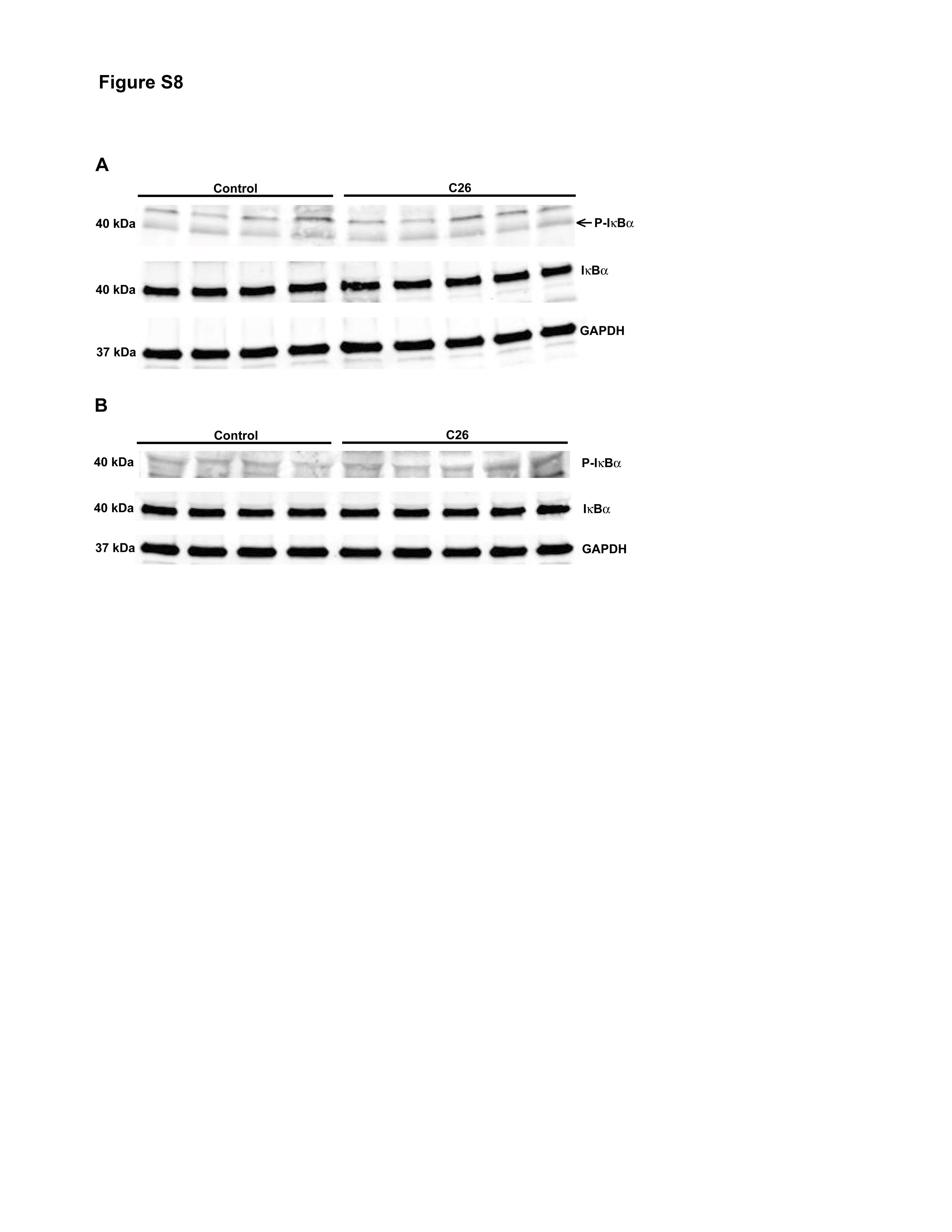

Supplement: Figure S8 — Western blots of phospho-IκBα and IκBα in control and C26 muscle. (A) Gastrocnemius muscle extracts from mice 10 days post inoculation. (B) Gastrocnemius muscle extracts from mice 24 days post inoculation. 60 µg protein loaded per lane. Each lane represents an independent muscle sample. There were no changes in p-IκBα or IκBα expression due to either time point of cachexia. (TIF) [file pone.0087776.s008.tif]
