# Supplementary material for: C26 Cancer-Induced Muscle Wasting Is IKKβ-Dependent and NF-kappaB-Independent
Source: PLoS One. 2014 Jan 29;9(1):e87776. doi: 10.1371/journal.pone.0087776 (PMC3906224; doi:10.1371/journal.pone.0087776)
Supplement: Figure S5 — FastQC printout for bam format of Control p65 ChIP-seq. The sequences passed all tests and were taken for peak analysis. (PDF) [file pone.0087776.s005.pdf]

FastQC Report

Mon 7 Oct 2013
Galaxy162-[106108.bam].bam

Summary

- Basic Statistics
- Per base sequence quality
- Per sequence quality scores
- Per base sequence content
- Per base GC content
- Per sequence GC content
- Per base N content
- Sequence Length Distribution
- Sequence Duplication Levels
- Overrepresented sequences
- Kmer Content

Basic Statistics

Measure	Value
Filename	Galaxy162-[106108.bam].bam
File type	Conventional base calls
Encoding	Sanger / Illumina 1.9

Total Sequences	10778354
Filtered Sequences	0
Sequence length	35-36
%GC	42

Per base sequence quality

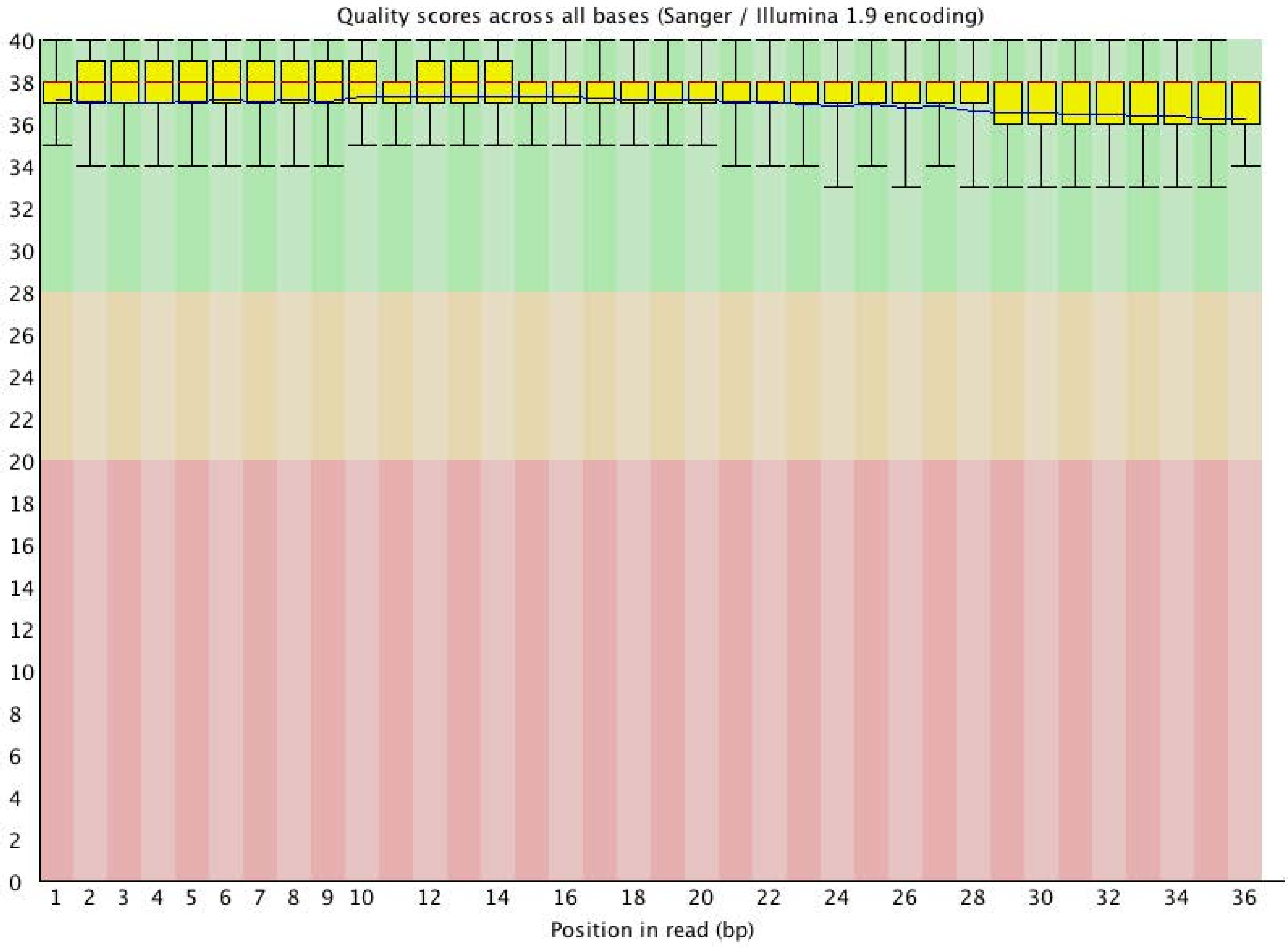
Quality scores across all bases (Sanger / Illumina 1.9 encoding)
40
38
36
34
32
30
28
26
24
22
20
18
16
14
12
10
8
6
4
2
0
1 2 3 4 5 6 7 8 9 10 12 14 16 18 20 22 24 26 28 30 32 34 36
Position in read (bp)

Per sequence quality scores

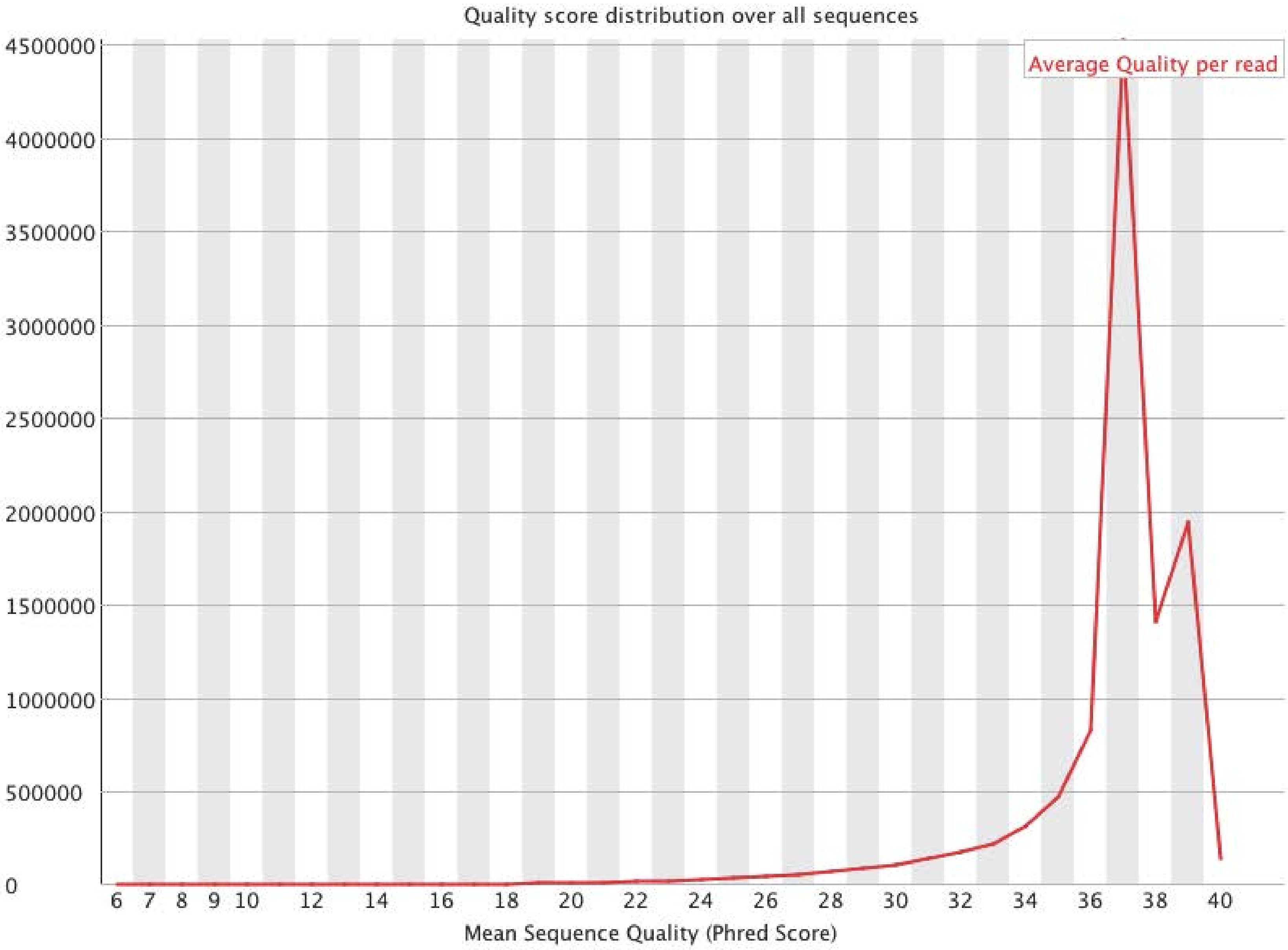

Per base sequence content

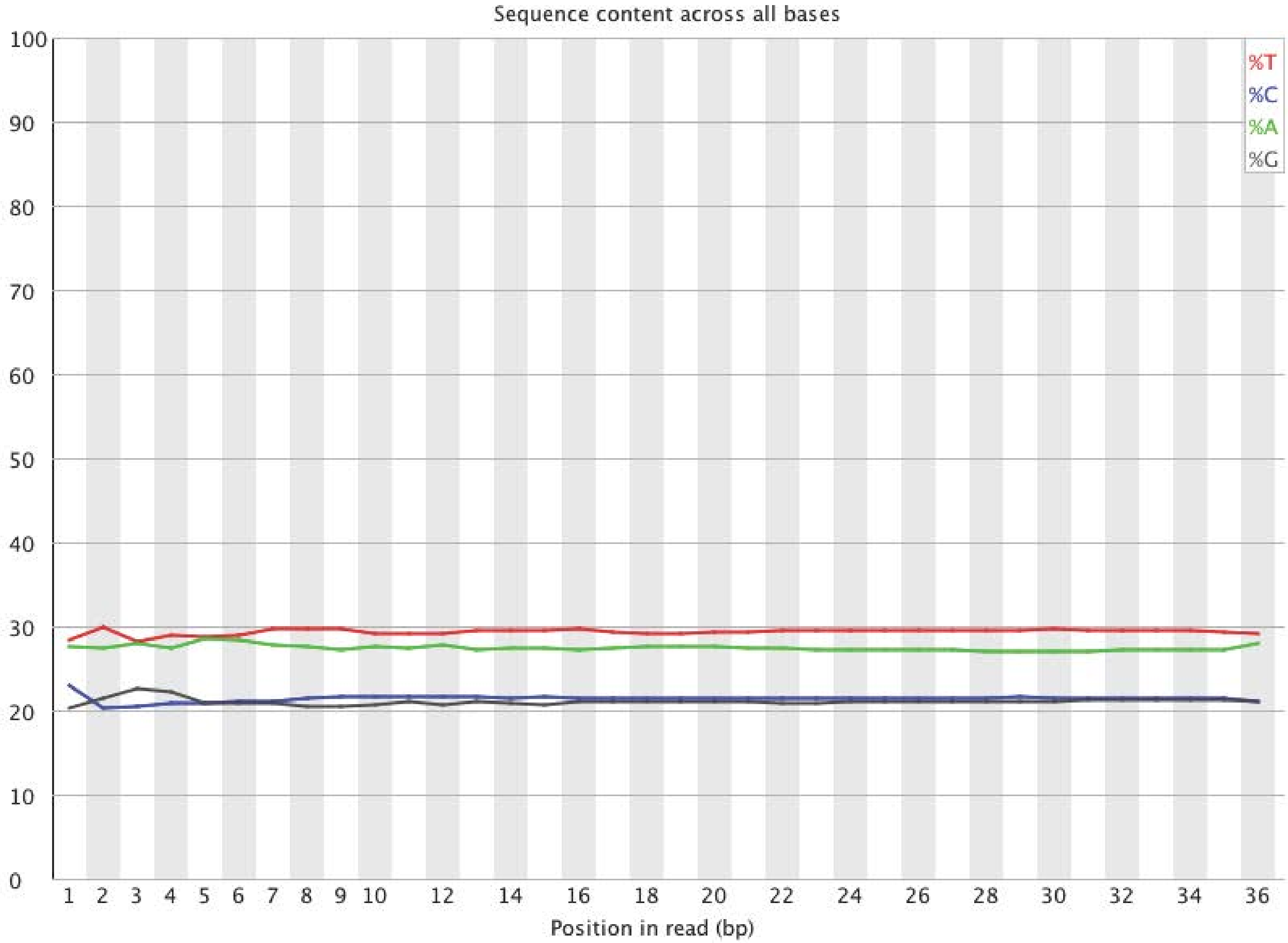

Per base GC content

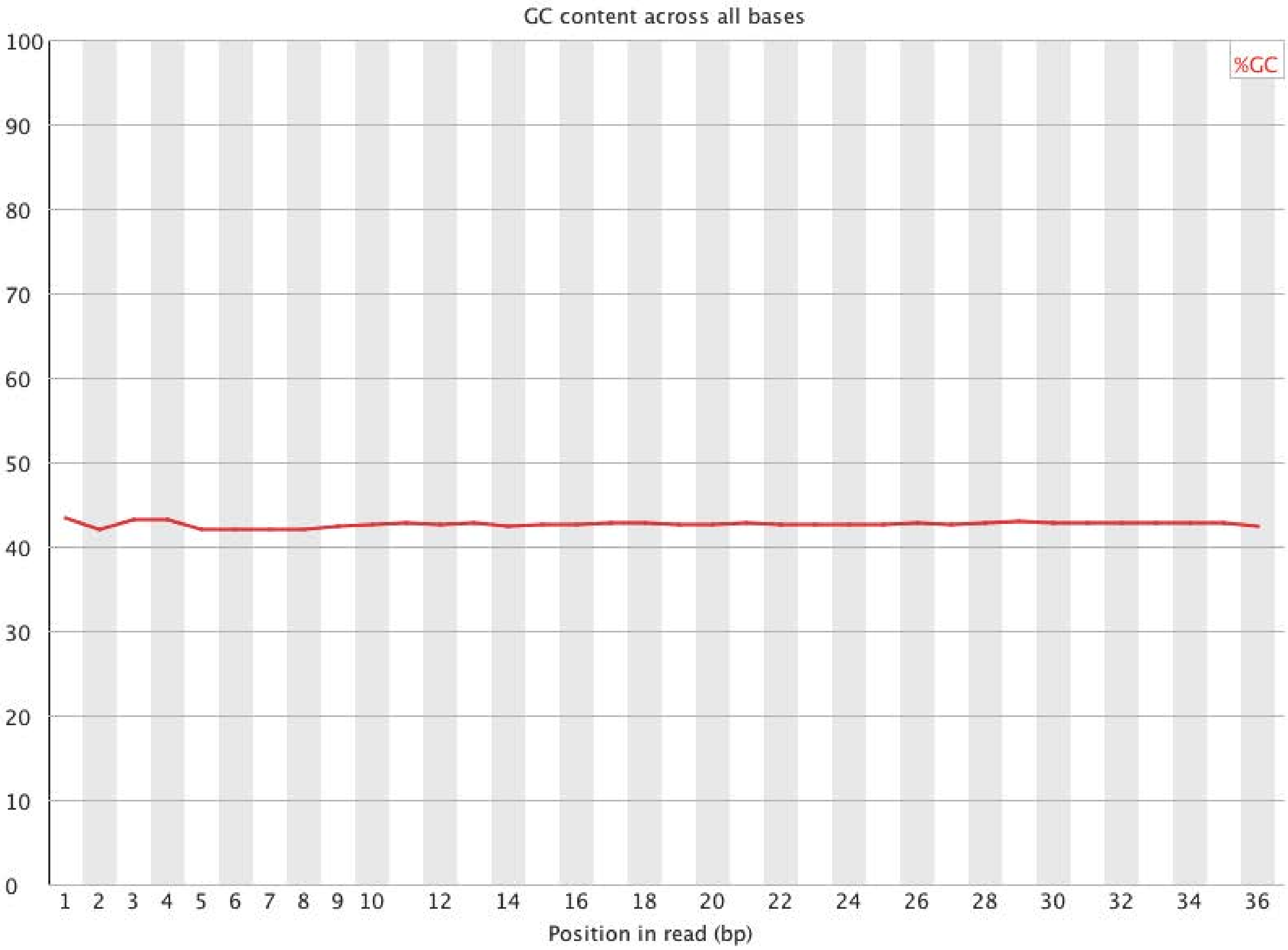

Per sequence GC content

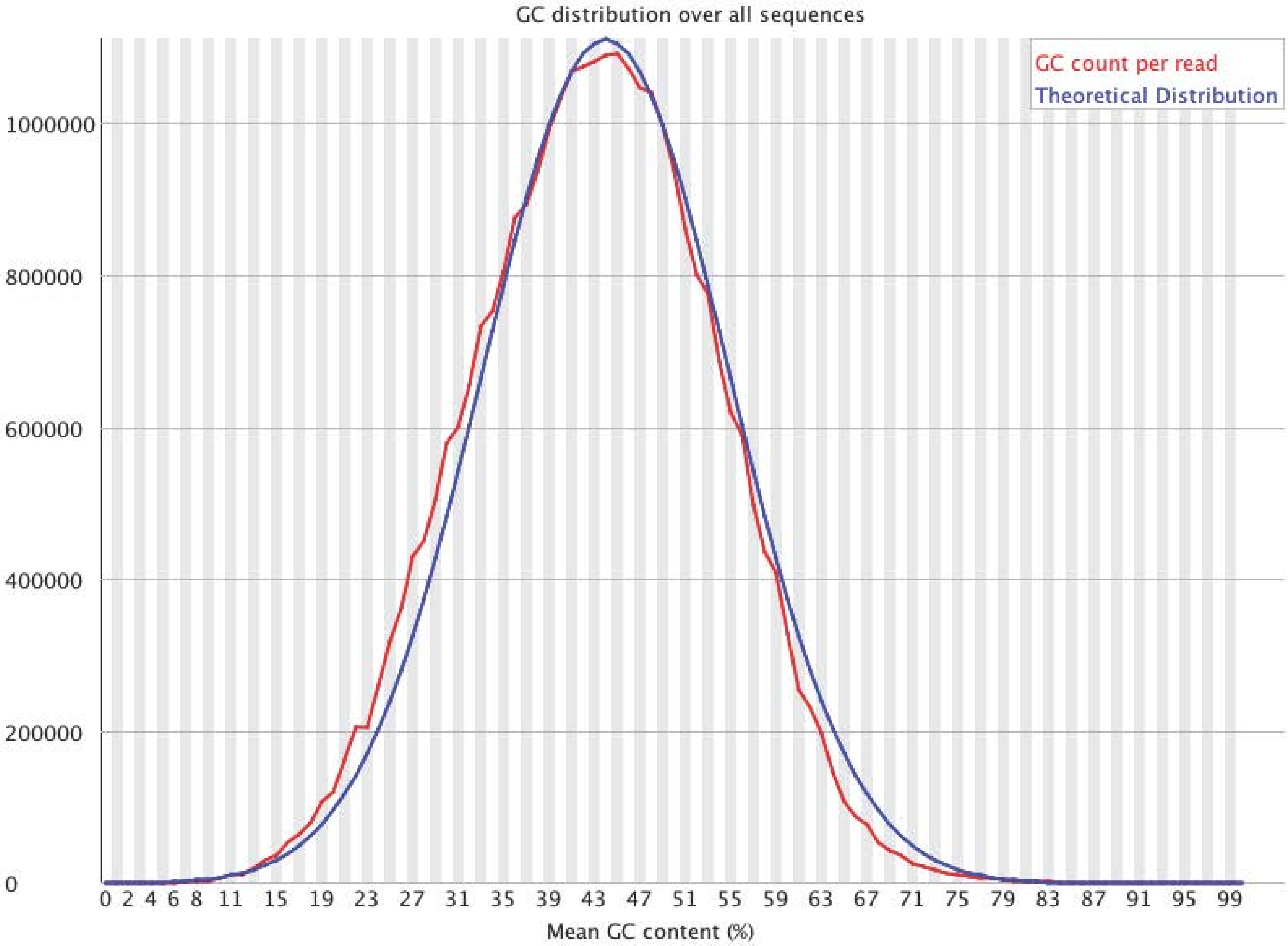

Per base N content

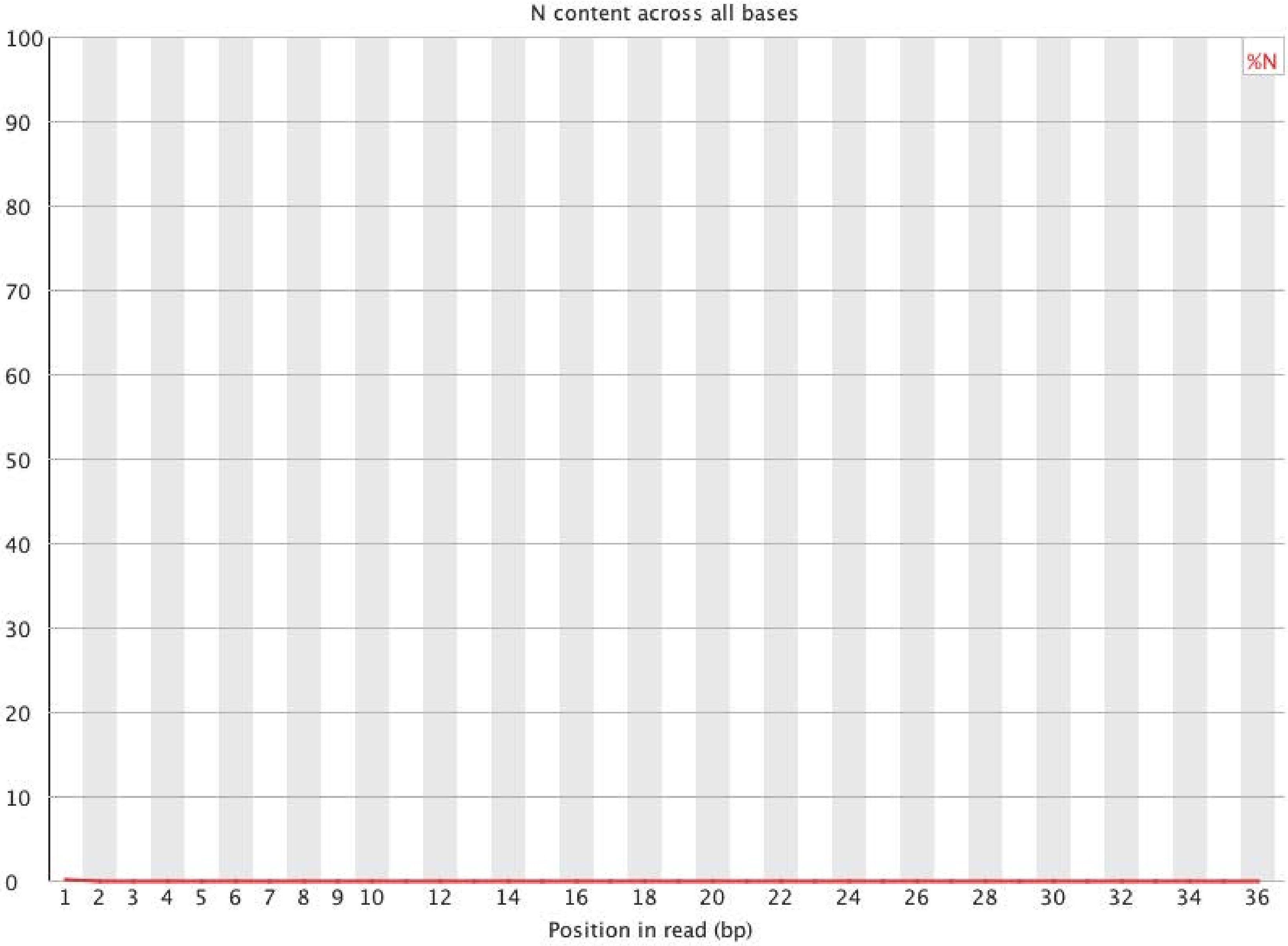

Sequence Length Distribution

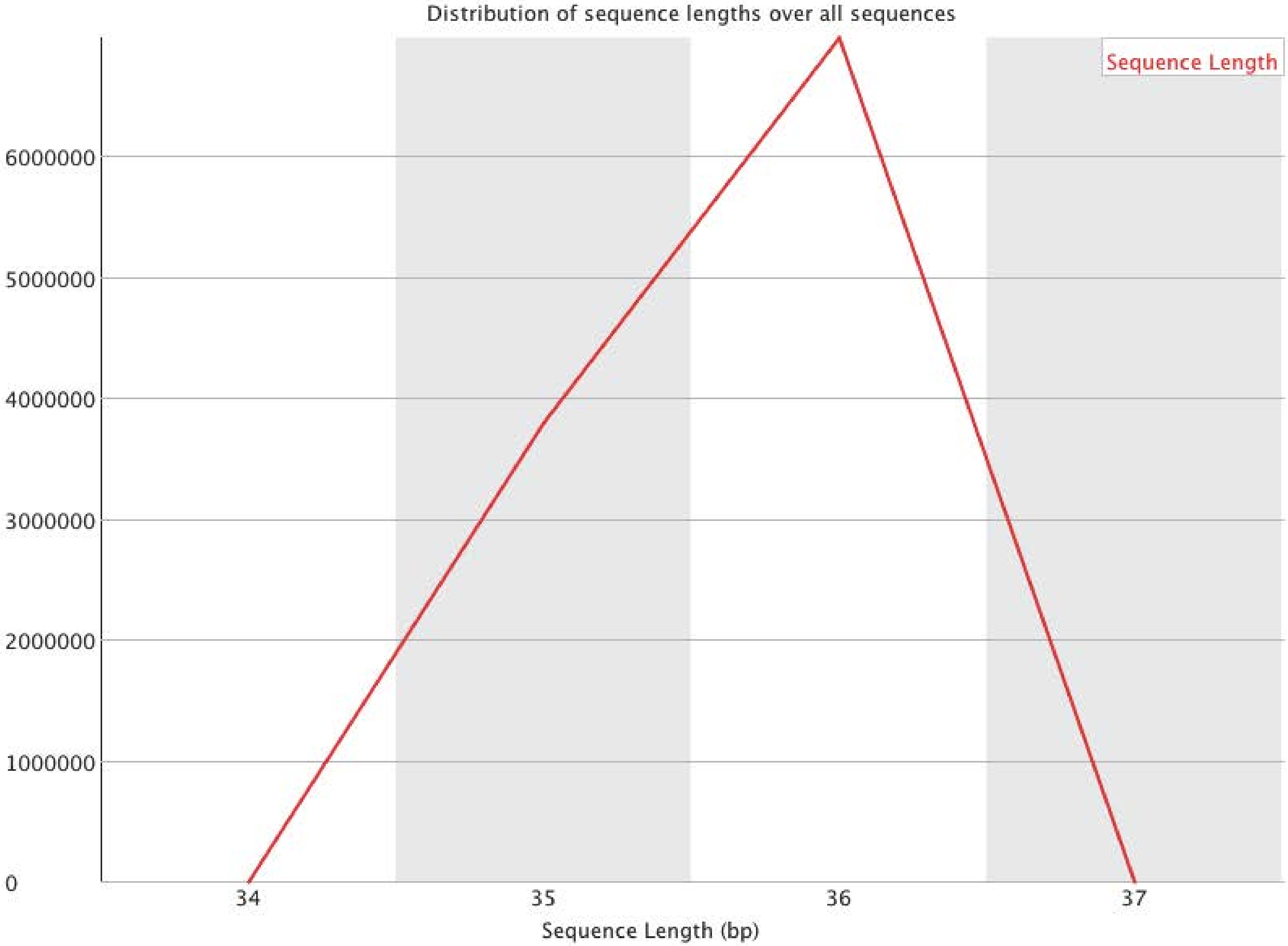

Sequence Duplication Levels

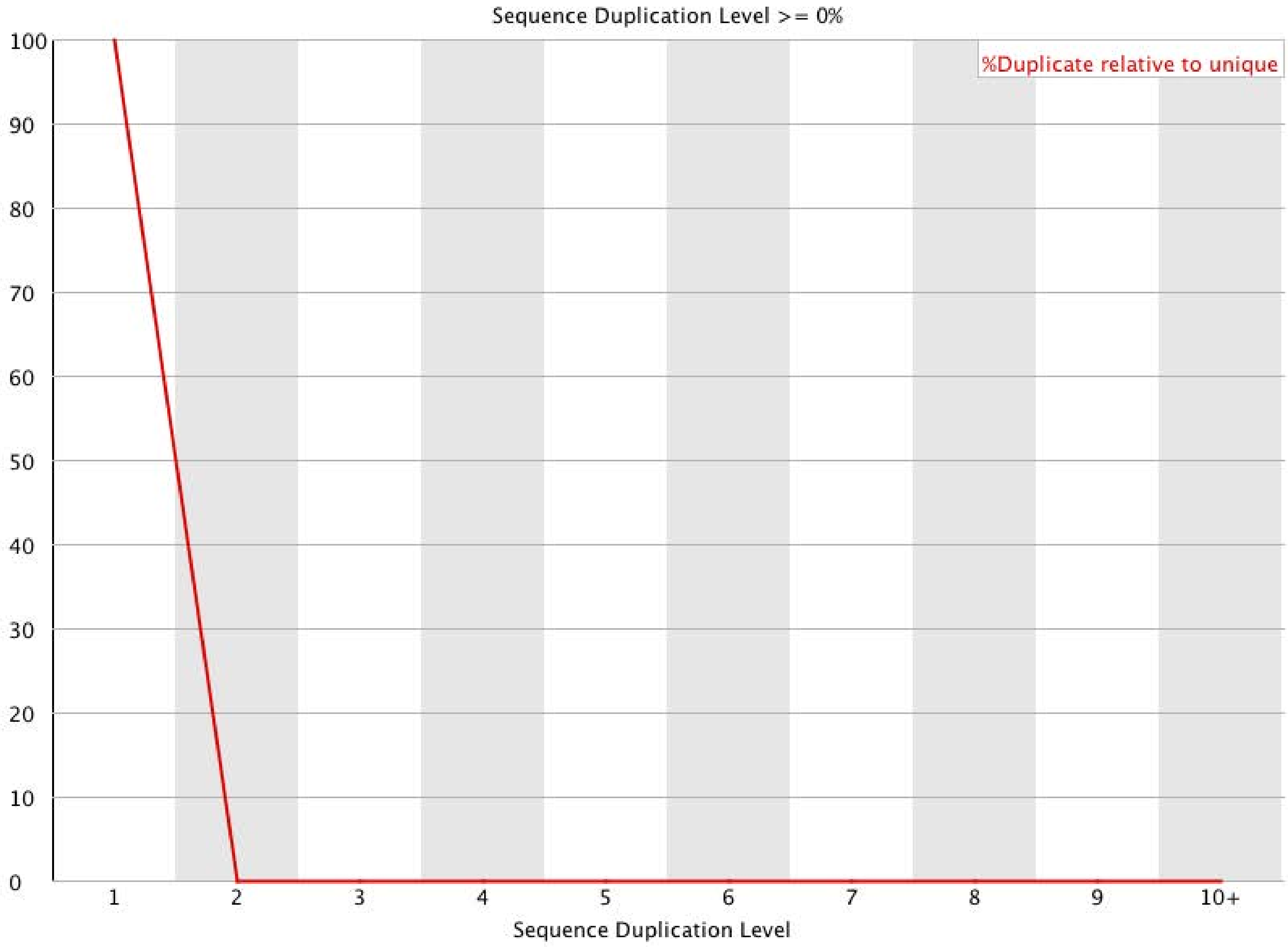
Sequence Duplication Level >= 0%
%Duplicate relative to unique
100
90
80
70
60
50
40
30
20
10
0
1
2
3
4
5
6
7
8
9
10+
Sequence Duplication Level

Overrepresented sequences

No overrepresented sequences

Kmer Content

No overrepresented Kmers

Produced by FastQC (version 0.10.1)
