# Supplementary material for: C26 Cancer-Induced Muscle Wasting Is IKKβ-Dependent and NF-kappaB-Independent
Source: PLoS One. 2014 Jan 29;9(1):e87776. doi: 10.1371/journal.pone.0087776 (PMC3906224; doi:10.1371/journal.pone.0087776)
Supplement: Table S3 — The genes with p65 ChIP-seq peaks from control (no tumor) and C26 muscle. From left to right the columns are: control p65, with reference to no antibody background alignments; C26 p65, with reference to no antibody background alignments; Intersect, the genes in common between control and C26; Control/Array+, the genes from Control ChIP-seq in common with >1.5 fold upregulated genes from the gene expression microarray; Control/Array-, the genes from the Control ChIP-seq in common with >1.5 fold downregulated genes from the gene expression microarray; C26/Array+, the genes from C26 ChIP-seq in common with >1.5 fold upregulated genes from the gene expression microarray; C26/Array-, the genes from the C26 ChIP-seq in common with >1.5 fold downregulated genes from the gene expression microarray. (DOCX) [file pone.0087776.s011.docx]

Table S3. The genes with peaks from p65 ChIP-seq

| **Control p65** | **C26 p65** | **Intersect** | **Control/Array+** | **Control/Array-** | **C26/Array+** | **C26/Array-** |
| --- | --- | --- | --- | --- | --- | --- |
| Ankrd11 | Akr1c21 | CAV2 | CBLB | HDHD3 | FBXO6 | AR |
| Bcl2l11 | Ano6 | CD200 | FKBP5 | IBTK |  | RHOBTB1 |
| Bcorl1 | Ar | EZH2 | FOSL2 | ITGB6 |  |  |
| Camk2g | Arhgap24 | GM15319 | HSF2 | JPH2 |  |  |
| Cav2 | Brd3 | GM17267 | MID1 | NAV2 |  |  |
| Cblb | Cav2 | GM17379 | STEAP4 |  |  |  |
| Cd200 | Cd200 | GM17467 |  |  |  |  |
| Cd244 | Cwc27 | GM17521 |  |  |  |  |
| Cdc27 | Cytip | GM4868 |  |  |  |  |
| Chrm2 | Dcaf4 | GM6604 |  |  |  |  |
| Cnga2 | Dux | GM773 |  |  |  |  |
| Col4a6 | Ezh2 | HEATR7B1 |  |  |  |  |
| Cxcl2 | Fbxo6 | HJURP |  |  |  |  |
| Cyth1 | Gm13212 | HSF2 |  |  |  |  |
| Dst | Gm15319 | MEX3C |  |  |  |  |
| Eepd1 | Gm17267 | MID1 |  |  |  |  |
| Efcab9 | Gm17379 | OSBPL10 |  |  |  |  |
| Efna5 | Gm17467 | SLC7A15 |  |  |  |  |
| Ehd4 | Gm17521 | SP110 |  |  |  |  |
| Epas1 | Gm2964 | SP140 |  |  |  |  |
| Ezh2 | Gm4868 | TRPM8 |  |  |  |  |
| Fkbp5 | Gm595 | UGT1A1 |  |  |  |  |
| Fosl2 | Gm6604 | VMN2R121 |  |  |  |  |
| Gm10719 | Gm773 | XLR |  |  |  |  |
| Gm12824 | Gm9867 | ZFP36L3 |  |  |  |  |
| Gm15319 | Heatr7b1 |  |  |  |  |  |
| Gm15319 | Hjurp |  |  |  |  |  |
| Gm15319 | Hsf2 |  |  |  |  |  |
| Gm17267 | Inpp5a |  |  |  |  |  |
| Gm17267 | Kcnip4 |  |  |  |  |  |
| Gm17379 | Lrch2 |  |  |  |  |  |
| Gm17467 | Mex3c |  |  |  |  |  |
| Gm17503 | Mid1 |  |  |  |  |  |
| Gm17521 | Ncor2 |  |  |  |  |  |
| Gm4868 | Osbpl10 |  |  |  |  |  |
| Gm6604 | Pdzd2 |  |  |  |  |  |
| Gm6604 | Pomc |  |  |  |  |  |
| Gm6793 | Pum1 |  |  |  |  |  |
| Gm773 | Rhobtb1 |  |  |  |  |  |
| Gna14 | Sema5a |  |  |  |  |  |
| Hdhd3 | Slc7a15 |  |  |  |  |  |
| Heatr7b1 | Sp110 |  |  |  |  |  |
| Hjurp | Sp140 |  |  |  |  |  |
| Hsf2 | Srpk2 |  |  |  |  |  |
| Ibtk | Tada3 |  |  |  |  |  |
| Ifi203 | Tnfsf15 |  |  |  |  |  |
| Ift27 | Trpm8 |  |  |  |  |  |
| Ikbkb | Ube2s |  |  |  |  |  |
| Itgb6 | Ugt1a1 |  |  |  |  |  |
| Itln1 | Ugt3a1 |  |  |  |  |  |
| Jph1 | Usp15 |  |  |  |  |  |
| Jph2 | Usp45 |  |  |  |  |  |
| Kcnma1 | Vmn2r121 |  |  |  |  |  |
| Kif26b | Vmn2r75 |  |  |  |  |  |
| Klf8 | Xlr |  |  |  |  |  |
| Lcp2 | Zfp36l3 |  |  |  |  |  |
| Lig1 | Zmiz1 |  |  |  |  |  |
| Lmbrd1 |  |  |  |  |  |  |
| Lpar3 |  |  |  |  |  |  |
| Mex3c |  |  |  |  |  |  |
| Mid1 |  |  |  |  |  |  |
| Mrpl32 |  |  |  |  |  |  |
| Nav2 |  |  |  |  |  |  |
| Olfm3 |  |  |  |  |  |  |
| Osbpl10 |  |  |  |  |  |  |
| Osbpl1a |  |  |  |  |  |  |
| Osbpl9 |  |  |  |  |  |  |
| Pank1 |  |  |  |  |  |  |
| Pisd |  |  |  |  |  |  |
| Rdh13 |  |  |  |  |  |  |
| Rnf128 |  |  |  |  |  |  |
| Rps26-ps1 |  |  |  |  |  |  |
| Scaf11 |  |  |  |  |  |  |
| Scd3 |  |  |  |  |  |  |
| Sfi1 |  |  |  |  |  |  |
| Sfi1 |  |  |  |  |  |  |
| Slc7a15 |  |  |  |  |  |  |
| Sp110 |  |  |  |  |  |  |
| Sp140 |  |  |  |  |  |  |
| Stag1 |  |  |  |  |  |  |
| Steap4 |  |  |  |  |  |  |
| Tmem39a |  |  |  |  |  |  |
| Tnfaip8 |  |  |  |  |  |  |
| Trim33 |  |  |  |  |  |  |
| Trim52 |  |  |  |  |  |  |
| Trpm8 |  |  |  |  |  |  |
| Ubr4 |  |  |  |  |  |  |
| Ugt1a1 |  |  |  |  |  |  |
| Usp32 |  |  |  |  |  |  |
| Vmn1r2 |  |  |  |  |  |  |
| Vmn2r121 |  |  |  |  |  |  |
| Vmn2r121 |  |  |  |  |  |  |
| Vmn2r121 |  |  |  |  |  |  |
| Vmn2r89 |  |  |  |  |  |  |
| Xlr |  |  |  |  |  |  |
| Zfp36l3 |  |  |  |  |  |  |
| Zfp512 |  |  |  |  |  |  |
| Zfp653 |  |  |  |  |  |  |
| Zfp692 |  |  |  |  |  |  |
| Zmat2 |  |  |  |  |  |  |
